# Opportunistic experiments to constrain aerosol effective radiative forcing

**DOI:** 10.5194/acp-22-641-2022

**Published:** 2022-01-17

**Authors:** Matthew W. Christensen, Andrew Gettelman, Jan Cermak, Guy Dagan, Michael Diamond, Alyson Douglas, Graham Feingold, Franziska Glassmeier, Tom Goren, Daniel P. Grosvenor, Edward Gryspeerdt, Ralph Kahn, Zhanqing Li, Po-Lun Ma, Florent Malavelle, Isabel L. McCoy, Daniel T. McCoy, Greg McFarquhar, Johannes Mülmenstädt, Sandip Pal, Anna Possner, Adam Povey, Johannes Quaas, Daniel Rosenfeld, Anja Schmidt, Roland Schrödner, Armin Sorooshian, Philip Stier, Velle Toll, Duncan Watson-Parris, Robert Wood, Mingxi Yang, Tianle Yuan

**Affiliations:** 1 Atmospheric, Oceanic and Planetary Physics, Department of Physics, University of Oxford, Oxford, OX1 3PU, UK; 2 Atmospheric Science & Global Change Division, Pacific Northwest National Laboratory, Richland, WA 99354, Washington, USA; 3 National Center for Atmospheric Research, Boulder, CO, USA; 4 Karlsruhe Institute of Technology (KIT), Institute of Meteorology and Climate Research, Karlsruhe, Germany; 5 Karlsruhe Institute of Technology (KIT), Institute of Photogrammetry and Remote Sensing, Karlsruhe, Germany; 6 Institute of Earth Sciences, The Hebrew University of Jerusalem, Jerusalem, Israel; 7 Department of Atmospheric Sciences, University of Washington, Seattle, USA; 8 NOAA Chemical Sciences Laboratory (CSL), Boulder, Colorado, USA; 9 Cooperative Institute for Research in Environmental Sciences (CIRES), University of Colorado, Boulder, Colorado, USA; 10 Department Geoscience and Remote Sensing, Delft University of Technology, P.O. Box 5048, 2600GA Delft, the Netherlands; 11 Institute for Meteorology, Universität Leipzig, Leipzig, Germany; 12 National Centre for Atmospheric Sciences, School of Earth and Environment, University of Leeds, Leeds, LS2 9JT, UK; 13 Space and Atmospheric Physics Group, Imperial College London, London, UK; 14 Earth Science Division, NASA Goddard Space Flight Center, Greenbelt, MD, USA; 15 Department of Atmospheric and Oceanic Science, University of Maryland, College Park, USA; 16 Met Office, Atmospheric Dispersion and Air Quality, Fitzroy Rd, Exeter, EX1 3PB, UK; 17 Rosenstiel School of Marine and Atmospheric Science, University of Miami, Miami, FL, USA; 18 Cooperative Programs for the Advancement of Earth System Science (CPAESS), University Corporation for Atmospheric Research, Boulder, CO, USA; 19 Department of Atmospheric Sciences, University of Wyoming, Laramie, USA; 20 Cooperative Institute for Severe and High Impact Weather Research and Operations (CIWRO) and School of Meteorology, University of Oklahoma, Norman, OK, USA; 21 School of Meteorology, University of Oklahoma, Norman, OK, USA; 22 Department of Geosciences, Texas Tech University, Lubbock, TX, USA; 23 Institute for Atmospheric and Environmental Sciences, Goethe University Frankfurt, Frankfurt am Main, Germany; 24 National Centre for Earth Observation, University of Oxford, Oxford, OX1 3PU, UK; 25 Department of Geography, University of Cambridge, Cambridge, UK; 26 Department of Chemistry, University of Cambridge, Cambridge, UK; 27 Leibniz Institute for Tropospheric Research, Leipzig, Germany; 28 Department of Chemical and Environmental Engineering, University of Arizona, Tucson, AZ, USA; 29 Department of Hydrology and Atmospheric Sciences, University of Arizona, Tucson, AZ, USA; 30 Institute of Physics, University of Tartu, Tartu, Estonia; 31 Plymouth Marine Laboratory, Prospect Place, Plymouth, PL1 3DH, UK; 32 Joint Center for Earth Systems Technologies, University of Maryland, Baltimore County, Baltimore, MD, USA; 33 Earth Science Division, NASA Goddard Space Flight Center, Greenbelt, MD, USA

## Abstract

Aerosol–cloud interactions (ACIs) are considered to be the most uncertain driver of present-day radiative forcing due to human activities. The nonlinearity of cloud-state changes to aerosol perturbations make it challenging to attribute causality in observed relationships of aerosol radiative forcing. Using correlations to infer causality can be challenging when meteorological variability also drives both aerosol and cloud changes independently. Natural and anthropogenic aerosol perturbations from well-defined sources provide “opportunistic experiments” (also known as natural experiments) to investigate ACI in cases where causality may be more confidently inferred. These perturbations cover a wide range of locations and spatiotemporal scales, including point sources such as volcanic eruptions or industrial sources, plumes from biomass burning or forest fires, and tracks from individual ships or shipping corridors. We review the different experimental conditions and conduct a synthesis of the available satellite datasets and field campaigns to place these opportunistic experiments on a common footing, facilitating new insights and a clearer understanding of key uncertainties in aerosol radiative forcing. Cloud albedo perturbations are strongly sensitive to background meteorological conditions. Strong liquid water path increases due to aerosol perturbations are largely ruled out by averaging across experiments. Opportunistic experiments have significantly improved process-level understanding of ACI, but it remains unclear how reliably the relationships found can be scaled to the global level, thus demonstrating a need for deeper investigation in order to improve assessments of aerosol radiative forcing and climate change.

## 1 Introduction

Numerous studies have attempted to quantify the different aerosol effects on warm liquid clouds. Increases in aerosol loading increase cloud drop number and decrease cloud drop size (the so-called Twomey effect; Twomey, 1974). However, microphysically driven adjustments in cloud properties like areal coverage and cloud water path that result from increased drop number remain uncertain. A reduction in precipitation due to smaller cloud droplets can moisten the atmosphere and enhance cloudiness (the so-called lifetime effect; Albrecht, 1989). At the same time, a larger number of smaller cloud droplets can also enhance the cloud-top evaporation and dry air entrainment (Wang et al., 2003) as well as reduce the sedimentation of cloud droplets (Bretherton et al., 2007), thereby leading to feedbacks which can decrease cloudiness. There is ample evidence of aerosol-driven precipitation suppression in stratocumulus (Wood et al., 2011), but the effects of this process on liquid water path and cloud fraction remain uncertain. Cloud adjustments may therefore either compound or counteract the cloud albedo (i.e., reflectance) change due to the “Twomey effect” of higher cloud droplet number and smaller droplet size. IPCC (2013) and Bellouin et al. (2020) confirmed these effects and their complexities using multiple lines of evidence. While mixed and ice phase clouds are critical to Earth’s radiation budget, and change in response to changing aerosol concentrations, we choose to focus on warm liquid clouds due to the wealth of existing knowledge and relative simplicity of this system.

A difficulty in understanding these aerosol–cloud interactions is that while it is easy to control experiments in model simulations (where aerosol populations are perturbed in a controlled manner in the same environment), this is not possible in the real world. Comparison of two different clouds with different aerosol populations requires understanding how the co-variability in aerosols and “meteorology” (defined as the temperature, specific humidity, turbulence, vertical motion, etc) affects cloud microphysical properties (liquid water path, drop number/size) and ultimately cloud radiative effects.

However, when emissions perturb aerosols in “controlled” (fixed or defined) conditions with minimal changes to meteorology, it is possible to use observations to understand ACI processes and to quantify the magnitude of anthropogenic aerosol radiative effects. These opportunistic experiments are defined as injections of aerosols into an environmental regime (“laboratory”) where the unperturbed state is to some extent known. While these are sometimes called “natural laboratories”, some are natural (e.g., volcanoes) while some are human-caused (e.g., industrial plumes and ship tracks). In this review, we will use the term “opportunistic” to apply to both. A laboratory refers to a regime (e.g., ship or volcanic emissions) and type of emission, while “experiment” refers to a particular case (e.g., a ship track or a shipping corridor).

The first type of experiment dates back to the 1960s when ship tracks, curvilinear cloud features that can be traced back to the movements of individual ships, were identified in Television Infrared Observation Satellite (TIROS) imagery of marine stratus decks (Conover, 1966). Since then several similar opportunistic experiments have been explored, such as aerosols emitted from industrial sources, volcanoes, and biomass burning plumes. Opportunistic experiments also include anthropogenic aerosol changes due to particular events, such as emission changes due to the 2008 Beijing Olympics, the “Great Recession” of 2007–2009, or even the COVID-19 pandemic. Finally, weekly cycles or long-term decadal trends have been used to understand aerosol radiative forcing and cloud modification on local to regional scales (Quaas, 2015).

This review will analyze different types of opportunistic experiments and how they can be used to test hypotheses about ACI and to quantify their effects to better constrain total anthropogenic aerosol forcing for warm boundary layer clouds. Some examples of mixed-phase, ice cloud, and convective cloud opportunistic experiments are discussed, but due to their episodic nature, heterogeneity of convection, and difficulty of detection, we primarily focus on warm cloud physics. Other examples such as aircraft contrails, dust events (e.g., Saharan air outbreaks), and cloud seeding to intentionally affect precipitation are also beyond the scope of this work.

The review is organized around two main types of opportunistic experiments covering different spatial scales. The first type is based on relatively small-scale perturbations in which “in-plume and out-of-plume” comparisons are possible (Sect. 2.1, 2.2, and to some extent 2.3 and 2.4). These cases provide opportunities to determine the unperturbed case (out-of-plume conditions), and thus, directly evaluating the response of clouds to aerosol perturbation under similar meteorological conditions. The second type of opportunistic experiments cover events with much larger spatial scales or comparing situations that are very distant in time (Sect. 2.5, 2.6, 2.7, and 2.8). In these cases the relevance to the climate scale is easier to establish, but it is much more difficult to determine the unperturbed/reference conditions. Then we provide a linked summary database of different experiments that have been used in previous studies (Sect. 3). Section 4 brings together the different experiment types to synthesize qualitative and quantitative aerosol effects across methods and experiment types. In Sect. 4 we also examine the factors controlling the cloud response to aerosol perturbations and the challenges of using small-scale perturbations to constrain ACI across spatiotemporal scales. Section 5 provides a synthesis of these findings and their conclusions.

## 2 Overview of opportunistic experiments

**Figure 1.**
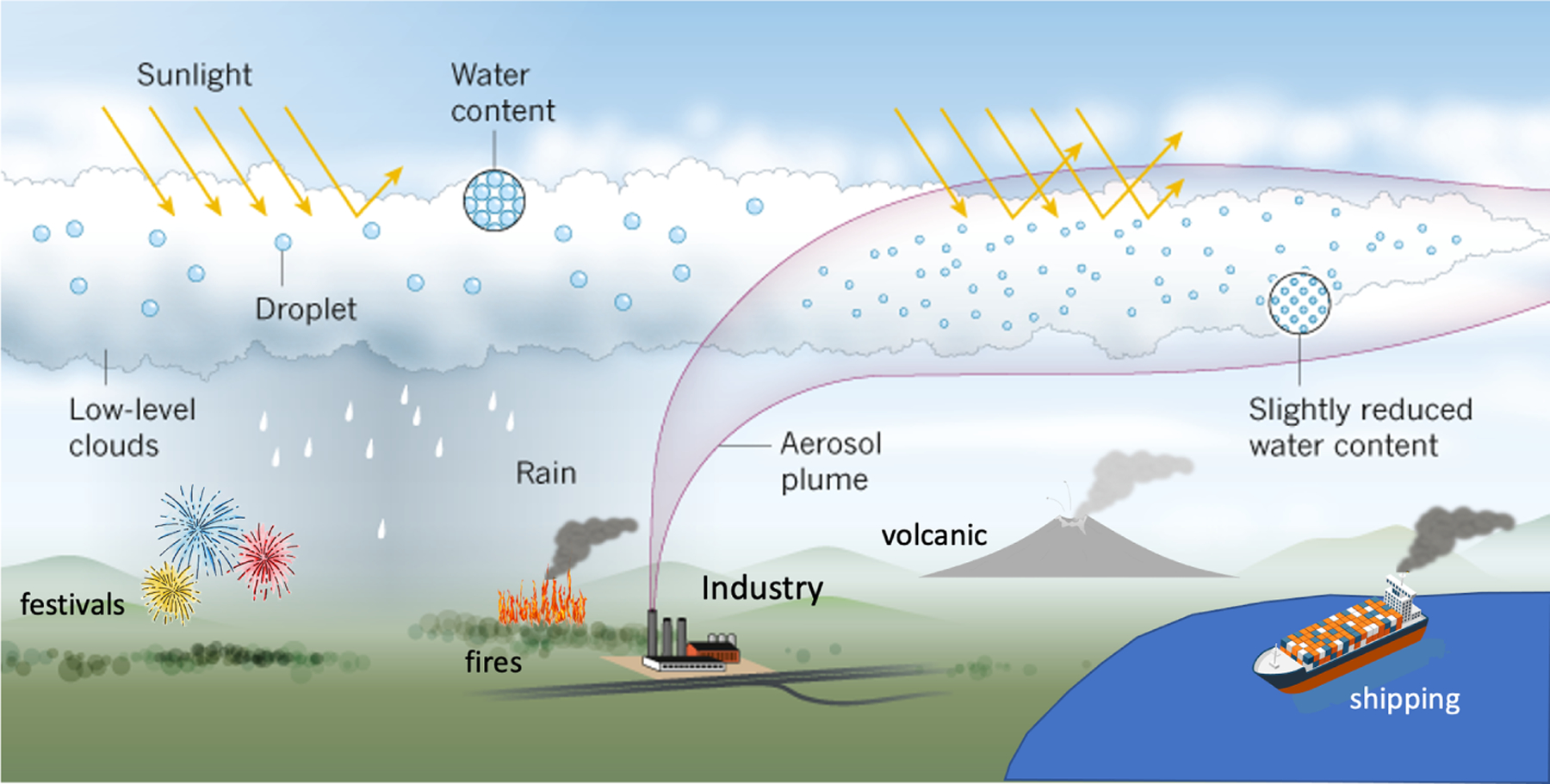
Schematic showing examples of the aerosol effect on boundary layer liquid clouds from some prominent natural laboratories found over the globe. Figure was adapted from Possner (2019).

Figure 1 highlights several key laboratories of significant interest and their influence on clouds and potentially climate. A wealth of papers describing cloud microphysical properties and their changes associated with each laboratory is described in Table S1 in the Supplement, and datasets generated for many of these papers are listed in Table S2. The following subsections provide a brief description of some of the primary characteristics of each laboratory and their strengths and limitations for teasing out process-level understanding of aerosol–cloud interactions.

**Figure 2.**
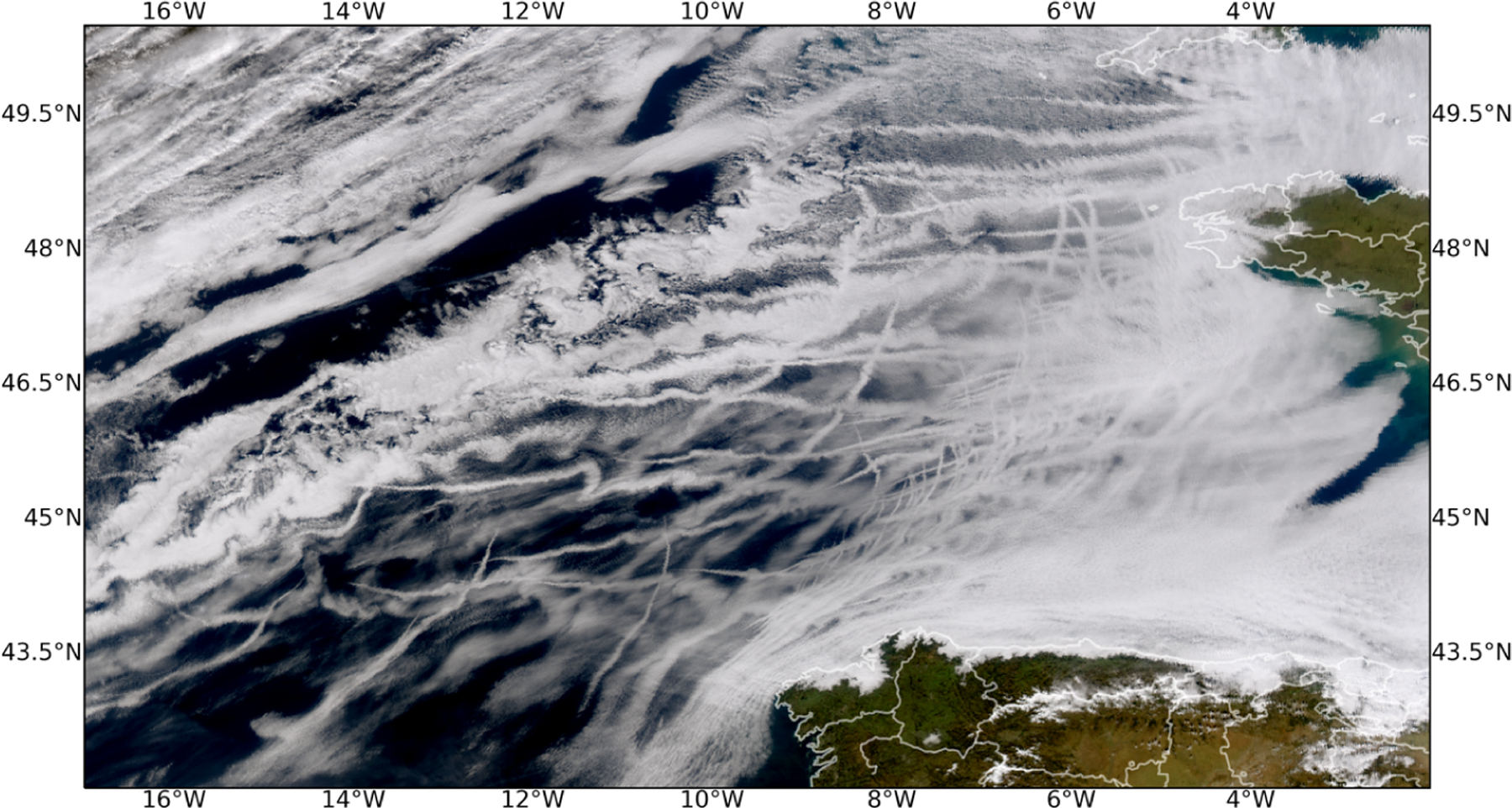
Ship tracks across the Bay of Biscay are shown in true color imagery from MODIS on the Aqua satellite on 27 January 2003 at 13:40 UTC.

**Figure 3.**
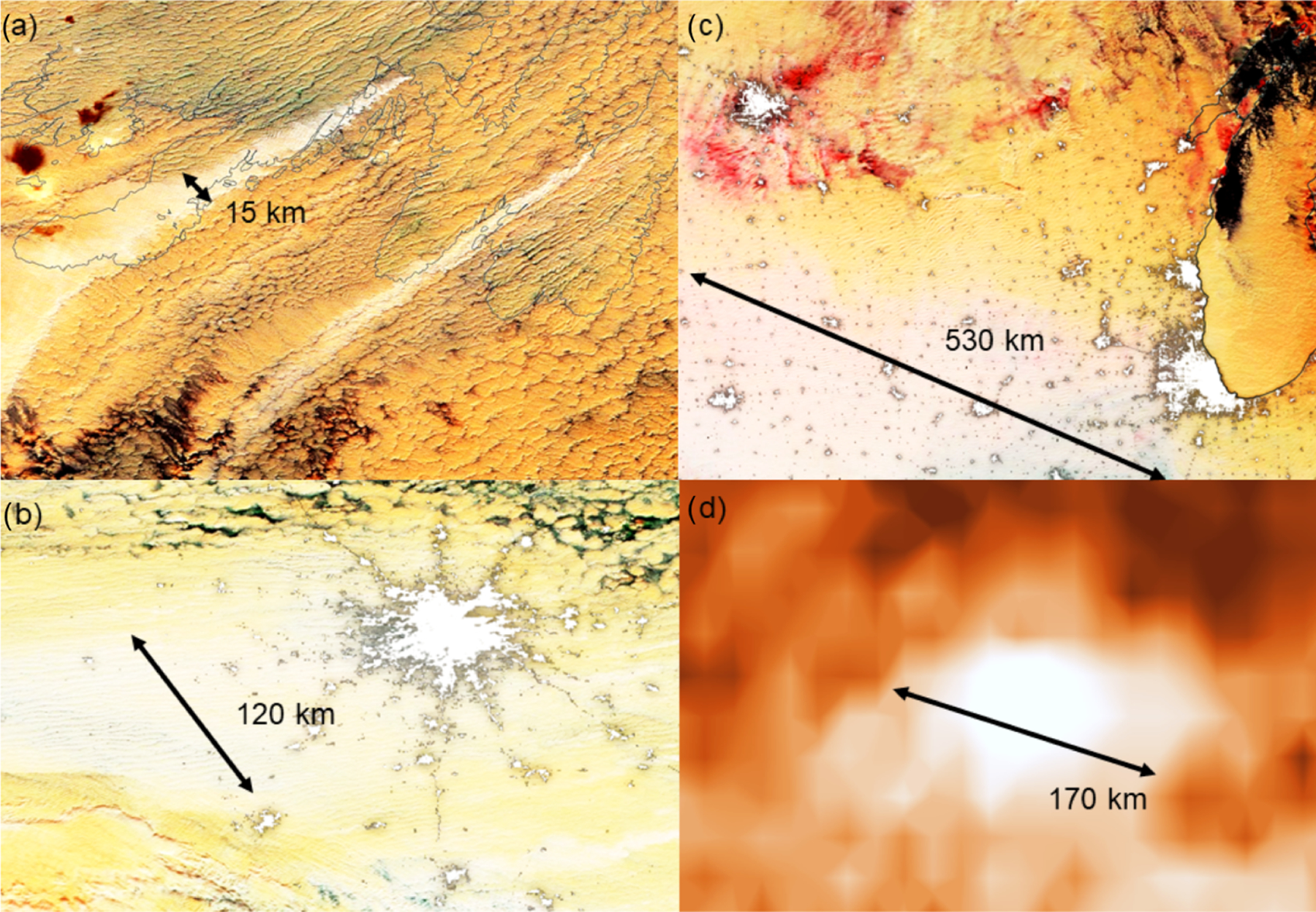
Polluted cloud tracks across spatial and temporal scales. Snapshot MODIS daytime near-infrared composite satellite images are shown in panels **(a–c)**: the polluted clouds are shown in bright greyish colors and unpolluted clouds in yellowish-brownish colors. Night lights are overlaid in white. **(a)** Two localized aerosol sources induce ship-track-like polluted cloud lines in Newfoundland, Canada, on 17 December 2014. The near-surface wind is blowing from the northeast based on MERRA reanalysis. **(b)** Emissions from Moscow, Russia, induce a more than 100 km wide polluted cloud area on 11 October 2016. Near the surface wind is blowing from the east based on MERRA reanalysis. **(c)** Many aerosol sources in the Great Lakes region, USA, induce a more than 500 km wide polluted cloud area on 3 January 2016. Near the surface wind is blowing from the northwest based on MERRA reanalysis. In panel **(d)** AVHRR cloud droplet effective radius data averaged over the years 1982 to 2015 are shown for the larger Moscow region. In the long-term average data, the cloud droplet effective radius decreases by 1 to 1.5 μm in the Moscow region compared to the nearby less polluted clouds. In panel **(d)** brownish colors represent larger droplets, and white colors represent smaller droplets.

Figure 2 shows an example of ship tracks off the coast of Portugal from the MODerate Resolution Imaging Spectroradiometer (MODIS) on the Aqua satellite. Generally, as the spatial domain of the aerosol perturbation increases (e.g., from individual ship tracks to shipping corridors to the entire globe) different methodologies are required to compute a counterfactual “background” (or unperturbed) cloud state. For example, in some experiments like ship, fire, and volcano tracks the counterfactual can easily be established by selecting unpolluted clouds in nearby locations in the same cloud regime. Establishing the observed counterfactual in opportunistic experiments involving large smoke plumes, volcanic eruptions, and shipping corridors is not as straightforward. The difficulty of attribution changes how effectively each opportunistic experiment can be studied and what kinds of conclusions can be drawn. Some prominent examples of natural laboratories and their associated opportunistic experiments (Fig. 3) are described below.

### 2.1 Shipping emissions

For decades, ships burning high-sulfur-content fuels have plied the world’s oceans, emitting aerosol and aerosolprecursor gases in regions with relatively low levels of natural aerosol (Capaldo et al., 1999; Eyring et al., 2010). The world’s major shipping routes have elevated concentrations of SO_2_ emissions according to the Emissions Database for Global Atmospheric Research (EDGAR), version 5.0 (Crippa et al., 2018, 2020), shown in Fig. S1 in the Supplement. Below we discuss some of the key opportunistic experiments: ship tracks, shipping corridors, and the role of policy change in ship emissions and associated radiative effects.

#### 2.1.1 Ship tracks

Ship tracks themselves have been studied since the mid-1960s, as soon as they were first identified in TIROS-VII imagery (Conover, 1966). The TIROS series was NASA’s first experiment with systematic satellite remote sensing of the Earth system. Multiple hypotheses, such as that the tracks were aircraft contrails or even secret missile tests, were considered before they were correctly identified as resulting from ships traveling through conditions of shallow, cloudy marine boundary layers (MBLs) with low background aerosol levels (Conover, 1966; Bowley, 1967; Twomey et al., 1968). In the late 1980s, satellite (Coakley et al., 1987) and aircraft (Radke et al., 1989) measurements confirmed the qualitative effects of ships on cloud properties hypothesized earlier. The apparent increase in liquid water content and decrease in drizzle-sized droplets in the ship tracks sampled by Radke et al. (1989) as well as cloud reductions along the edges of ship tracks from local-scale circulations (Scorer, 1987) served as a partial inspiration for the modeling work generally credited with establishing the cloud adjustment (“drizzle suppression”) hypothesis (Albrecht, 1989).

More systematic measurements of ship tracks were taken during the Monterey Area Ship Track experiment (MAST) campaign in the mid-1990s (Ackerman et al., 2000; Durkee et al., 2000b, a; Hobbs et al., 2000; Ferek et al., 2000). An analysis of 131 ship tracks studied in MAST showed that the tracks tended to form in shallow boundary layers (300–750 m) and last for 7 h on average, with many lasting longer than 12 h (Durkee et al., 2000a). Cloud condensation nuclei (CCN) emitted from the ships directly and potentially coated by sulfate (from the SO_2_ co-emitted with carbonaceous particles in fuel burning) were found to be responsible for influencing cloud properties, rather than any effects from sea salt produced in ships’ wakes or from the temperature or moisture perturbations associated with fuel burning (Durkee et al., 2000b; Hobbs et al., 2000). Several subsequent campaigns continued studying ship impacts on clouds, including the Marine Stratus/Stratocumulus Experiments (MASE) I and II (Lu et al., 2009), the Eastern Pacific Emitted Aerosol Experiment (E-PEACE) (Russell et al., 2013), and the Nucleation in California Experiment (NiCE) (Sorooshian et al., 2015). The majority of these campaigns were conducted using the Center for Interdisciplinary Remotely-Piloted Aircraft Studies (CIRPAS) Twin Otter aircraft, which would fly directly to ships and then conduct zigzag or racetrack patterns behind ships to characterize both the clean and perturbed boundary layer (Sorooshian et al., 2015, 2018). Relevant payload instruments included those measuring droplet size distributions, composition of both cloud water and droplet residual particles to chemically confirm evidence of ship influence, and aerosol size distributions and composition below cloud base. The data show clear evidence of clouds perturbed by ship plumes based on sharp enhancements in *N*_d_.

Some MAST observations did appear to support the lifetime effect hypothesis of Albrecht (1989), such as the finding that drizzle was generally reduced in ship tracks (Ferek et al., 2000). However, a weak anti-correlation was observed between liquid water content and cloud droplet number concentration (*N*_d_) within a sample of 69 ship tracks (Ackerman et al., 2000). Later satellite analyses of ship tracks also cast doubt on a unidirectional lifetime effect by demonstrating that decreased liquid water path (LWP) within ship tracks was a frequent occurrence (Coakley and Walsh, 2002). In approximately 30 % of cases, this decrease in LWP is enough to offset the brightening from the Twomey effect entirely and actually darken the ship tracks (Chen et al., 2012). While darkened ship tracks can occur in satellite imagery (Fig. S2), ship tracks (particularly those forming in typical closed-cell stratocumulus) sometimes lack a sufficient signal-to-noise ratio in the near-infrared reflectance between the polluted and surrounding unpolluted clouds, which can be a significant issue for estimating radiative forcing when the signal is relatively small (in contrast to the background clouds being highly noisy). Systematic studies of ship tracks from across many ocean basins suggest that ship emissions have a varied influence on LWP, with large increases occurring under clean conditions and decreases under more polluted conditions (Gryspeerdt et al., 2019b). However, the overall effect of LWP changes from all ship tracks has been estimated to be small compared to the relative changes in droplet number concentration on average (Toll et al., 2019). In particular, LWP tends to increase when clouds are drizzling (as inferred from CloudSat observations) and are topped by a relatively moisture-free troposphere but decrease in non-precipitating and drier cases (Toll et al., 2017).

Many of the satellite observational studies use passive satellite imagers such as AVHRR (Advanced Very High Resolution Radiometer) and MODIS. The spatial resolutions are typically 1 km, making them very useful for detection and attribution, but they only provide imagery once per day from each platform. Geostationary satellites are an ideal tool for investigating time-dependent processes in response to aerosol (Goren and Rosenfeld, 2012, 2015; Christensen et al., 2020) because of their ability to take snapshots throughout the day, but their observations are often difficult to utilize due to uncertainty in their calibration, limited spectral coverage, and impractical data volumes. With the new high-resolution Advanced Baseline Imager (ABI) on the GOES and Himawari platforms, local-scale cloud retrievals can be performed within cloud fields perturbed by point-source emissions as the response evolves (Minnis et al., 2008).

Other studies have investigated potential increases in cloud top height from in situ aircraft measurements (Taylor and Ackerman, 1999) and lidar retrievals from satellite data (Christensen and Stephens, 2011) as well as differences in responses between closed-cell and open-cell mesoscale convective organization (Christensen and Stephens, 2012). Ship emissions can increase cloud fraction (*C*_F_) even without changing cell structure (Feingold et al., 2015). Furthermore, transitions from open-cellular to closed-cellular convection induced by ship emissions provide evidence of a cloud fraction enhancement occurring over several days following the evolution of several dozen ship tracks (Goren and Rosenfeld, 2012). These older, more diffuse ship tracks do not typically retain their original track-like characteristics, thereby making them difficult to detect without geostationary satellite observations and are thus underrepresented in nearly all ship track studies.

Instantaneous satellite observations from polar-orbiting satellites can be used to infer time-dependent processes. Gryspeerdt et al. (2021) used MODIS imagery to study ship track evolution, assuming that the response of clouds to the ship emissions as a function of time is related to the distance from the head of the ship track. This novel methodology allows the use of higher resolution, relative to most of the older generation geostationary satellites, to investigate aerosol–cloud interaction from satellites. Goren and Rosenfeld (2015) used geostationary satellite observations to relate long-lived extensive overcast stratocumulus deck to air pollution originating in western Europe. Complementing their satellite analysis with an aerosol transport model and in situ observations of CO concentration, they explicitly showed that a closed-cell cloud deck was associated with polluted continental outflow from western Europe.

Ship track studies, thus, have been very helpful in formulating and testing many hypotheses about aerosol–cloud interaction mechanisms. However, observational studies aiming to quantify the effects from shipping at climatically relevant temporal and spatial scales have tended to find negligible or undetectable effects (Schreier et al., 2007; Peters et al., 2011, 2014), at least until recently. Schreier et al. (2007) analyzed 1 year of manually detected ship tracks within low-cloud-dominated satellite scenes and calculated a negligible global radiative forcing of −0.0004 to −0.0006 Wm^−2^. However, a very large percentage of ship tracks likely go undetected, as there are on the order of 100 000 ships in the global fleet (Eyring et al., 2010), and yet studies of ship tracks tend to identify only hundreds to thousands of tracks per year (Campmany et al., 2009; Christensen et al., 2014; Toll et al., 2017, 2019; Gryspeerdt et al., 2019a). The under-identification of ship tracks is likely to be especially pronounced in more complex cloud scenes (Possner et al., 2018). This lack of detection may suggest that either our observing systems are not sensitive enough or methodologies are not sophisticated enough to capture the many weak ship track signatures. New automated methods for identifying ship tracks using machine learning (Yuan et al., 2019) or by following air mass trajectories to interpolate between observed ship track segments (Gryspeerdt et al., 2021) hold promise for identifying a substantially larger number of ship tracks than has previously been possible. An outstanding question is whether these weak tracks are frequent enough to have a noticeable effect on shortwave reflection to space. However, because the tracks are weak, a large number of cases would be needed to contribute significantly to the radiation budget. In a global modeling study, Peters et al. (2013) showed significant radiative effects (0.3 Wm^−2^) from the net emissions of global shipping. Contrasting their results to the satellite observations suggests that the integrated radiative effect from easily detected and isolated ship tracks make up a small contribution to the total aerosol indirect radiative effect from shipping.

Liu et al. (2000) performed numerical modeling experiments of ship emissions and found that boundary layer decoupling is an important process that affects the vertical transport of ship emissions. Berner et al. (2015) used large-eddy simulation (LES) to simulate a particular observed ship-track case and demonstrated a good agreement with observations. LES sensitivity studies demonstrated the role of the alignment between the track and the winds in the boundary layer and of the ambient aerosol concentration in determining the magnitude of the response (Berner et al., 2015). Wang and Feingold (2009) used LES to study how emitted aerosols are transported within the marine boundary layer and how they impact cloud microphysical processes, and development. They also demonstrated that the amount of cloud brightening strongly depends on meteorology, background aerosol conditions, and the effect of secondary circulations (discussed in Sect. 4.7.1). Goren et al. (2019) further used LES in a Lagrangian setup, in which clouds are simulated along a realistic observed trajectory and are driven by meteorological conditions taken from reanalysis. They showed that closed cells, which formed within a polluted air mass, would have broken up sooner in a cleaner atmosphere. While aerosol was the main factor determining the consistent delayed cloud breakup by suppressing precipitation onset, the breakup time was also significantly modulated by LWP changes driven by diurnal cycle and large-scale meteorology.

#### 2.1.2 Shipping corridors

In order to evaluate shipping effects more holistically, several studies have attempted to circumvent issues involving detection and identification of individual ship tracks by analyzing entire shipping corridors instead. Peters et al. (2011) evaluated satellite-derived cloud properties upstream and downstream of three tropical and subtropical shipping corridors in which low-level winds typically blow perpendicular to the corridors, under the hypothesis that observations upstream of the corridor would represent unpolluted clouds and those downstream would show the effect of shipping pollution. No statistically significant impacts from shipping could be detected. However, Peters et al. (2011) lacked the control conditions against which to contrast the changes due to shipping. A follow-up analysis applying this same methodology to climate model output confirmed that natural sources of meteorological variability and gradients in cloud properties obscure the effects of shipping (Peters et al., 2014). Diamond et al. (2020) found substantial increases in climatological *N*_d_ and cloud reflectance within a shipping corridor in the southeast Atlantic Ocean, the primary difference from the earlier work being that low-level winds, parallel with the shipping corridor, keep the ship emissions relatively concentrated. They employed a method in which the cloud and aerosol properties within the corridor that would be expected to exist in the absence of shipping emissions (the “counterfactual” situation) were estimated via a universal kriging algorithm trained on nearby presumably non-shipping-affected values. The difference between the counterfactual and the observed or reanalysis cloud and aerosol properties (“factual”) was taken as the effect of shipping emissions.

**Figure 4.**
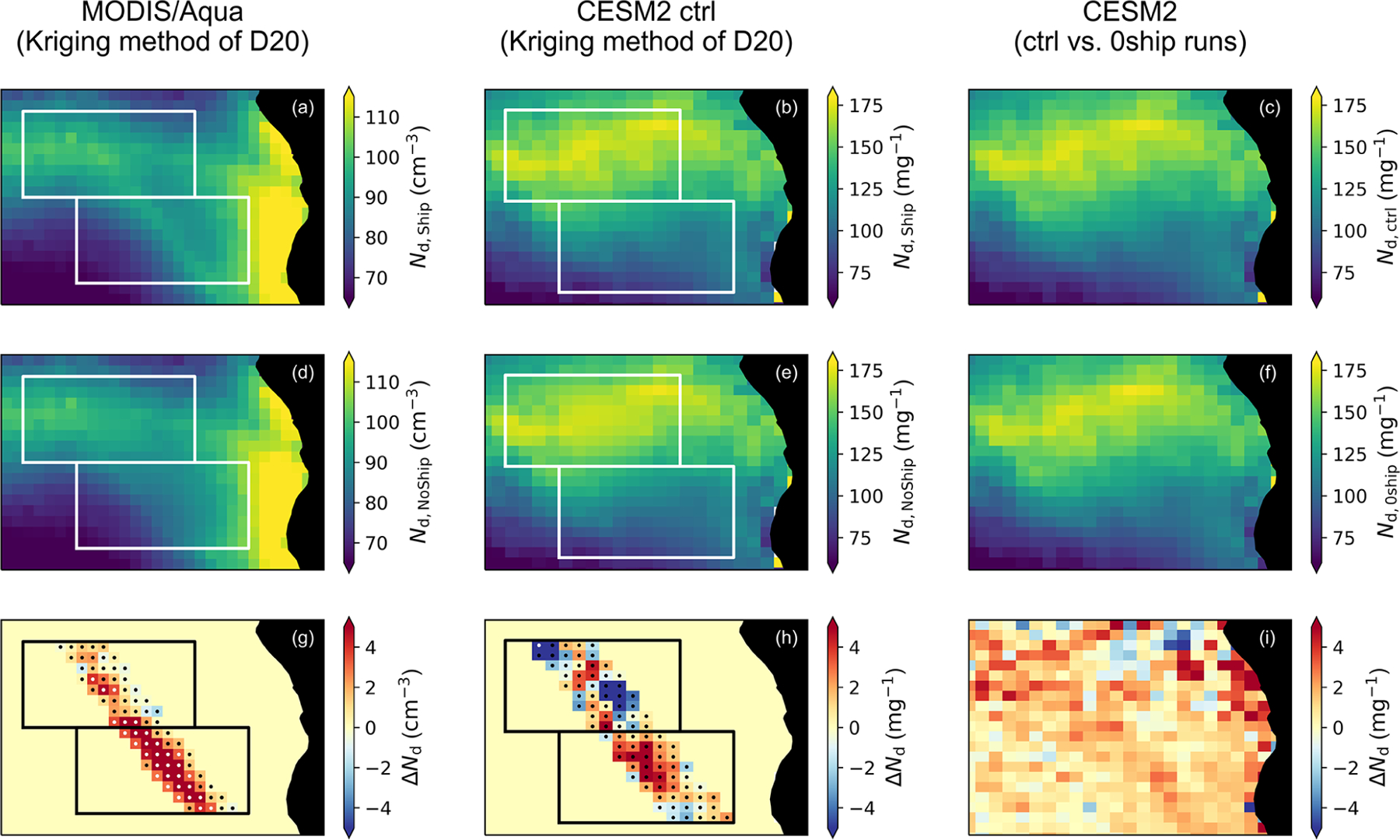
Comparison of Diamond et al. (2020) shipping corridor results for cloud droplet number concentration with CESM2 output. Factual (“Ship”) fields for **(a)** MODIS/Aqua and **(b, c)** CESM2 control (“ctrl”), counterfactual (“NoShip”) fields obtained by kriging for **(d)** MODIS/Aqua and **(e)** CESM2 ctrl and **(f)** results from CESM2 with zero shipping emissions (“0ship”), and the **(g, h)** factual–counterfactual or **(i)** ctrl–0ship differences. For panels **(g, h)**, white dots indicate significance at 95 % confidence, whereas black dots indicate values that are not statistically significant.

Figure 4 shows a comparison of the results from Diamond et al. (2020) for *N*_d_ with output from the Community Earth System Model version 2 (CESM2) analyzed in a similar manner (see Sect. S1 in the Supplement for full details). In contrast to the MODIS/Aqua observations (Fig. 4g), CESM2 does not show a clear, statistically significant enhancement in *N*_d_ coincident with the major southeast Atlantic shipping corridor (Fig. 4h). A similar analysis performed for surface sulfate mass concentration (Fig. S3) shows that there is a perturbation coincident with the shipping corridor as expected, albeit weaker and more diffuse than that inferred from the Modern-Era Retrospective analysis for Research and Applications, Version 2 (MERRA-2; Randles et al., 2017). However, one must recognize the uncertainty in sulfate evolution from SO_2_, including primary sulfate fractions and size distributions that could contribute to these differences. Comparing the control run of CESM2 (with normal shipping emissions included, Fig. 4c) with an experimental run with shipping emissions set to zero (Fig. 4f) shows that shipping emissions cause a broad increase in *N*_d_ over the southeast Atlantic with some hint of a particular enhancement within the heavily trafficked corridor (Fig. 4i). Similarly, Fig. S3 shows that the greatest enhancement in sulfate from shipping emissions occurs within the corridor but that there is a sizable effect throughout the entire region as well (as also found by Peters et al., 2014). Thus, comparisons of the observational and reanalysis-based results of Diamond et al. (2020) with climate model data may not be straightforward, and detailed cloud processes prove challenging to resolve. In part, this may be due to the much larger heterogeneity in the model mean cloud properties compared to the observations, in terms of both the overall spread in values and their smoothness in space. Climate model studies focused on comparing output to observed corridor perturbations may need to restrict emission reductions to the region of interest only, as opposed to reducing emissions worldwide, due to the non-negligible contributions from longer-range transport.

Another potential caveat to consider is that the presence of black carbon may lead to cloud burn-off and affect cloud properties in this shipping corridor off the coast of South Africa (Hu et al., 2021), although attempts to quantify this effect suggest its magnitude may be insignificant (Diamond et al., 2020). Also, ship emissions may also be important for mixed-phase cloud properties, although studies have suggested that the effect on cloud brightness is more muted than in warm clouds (Christensen et al., 2014; Possner et al., 2017). LES has also been shown to be useful for studying the response of mixed-phase clouds to ship emissions (Possner et al., 2017), and the commonalities to and differences between the response of mixed-phase and warm clouds have been demonstrated. Shipping may even affect deep convective clouds: lightning appears to be enhanced over major shipping corridors in the northeastern Indian Ocean and the South China Sea, which has been hypothesized to be due to convective invigoration from shipping-related aerosol perturbations in a well-defined shipping lane flanked by lower background aerosol concentrations (Thornton et al., 2017; Blossey et al., 2018; Grabowski and Morrison, 2020). However, the low aerosol baseline with more lightning in the shipping lane is also consistent with a signature of rainfall scavenging and does not imply causality.

#### 2.1.3 Global response and policy change

Cloud sensitivity to ship emissions on a larger, more climate relevant, scale is estimated using general circulation models (GCMs). For example, Lauer et al. (2007) used a GCM to study the impact of particulate matter from ship emissions on aerosols, clouds, and the radiation budget under different emission inventories. They demonstrated that emissions from ships increased the area mean *N*_d_ of low marine clouds by up to 30 % depending on the geographic region, while the change in liquid water content was small. In addition, the *R*_e_ values were shown to decrease, leading to an increase in cloud optical thickness of up to 5 %–10 %, again, depending on the geographical region. Jin et al. (2018) used a GCM to show that the cloud response to ship emissions depended on the natural dimethyl sulfide (DMS) emissions, which determine the background aerosol concentration and cloud sensitivity. In addition, they estimated the global net cloud radiative effect of ship emissions to be −0.153 Wm^−2^.

Another example of an opportunistic experiment recently manifested itself temporally through a policy change. On 1 January 2020, the International Maritime Organisation (IMO) of the United Nations mandated that for all ships, the maximum allowed sulfur content relative to mass of fuel needs to be reduced from 3.5 % to 0.5 %, hence reducing the amount of sulfur compounds emitted into the atmosphere. This was largely accomplished by burning lower sulfur fuel oil (the strategy employed thus far by ca. 90 % of the global fleet as of 2021) or installing scrubbers on ship exhaust (ca. 10 % of ships). In 2015, similar policy changes were carried out but only surrounding the US and European nearshore coastal regions called sulfur emission control areas (SECAs) near the US and European coasts (where only 0.1 % of sulfur in the fuel is allowed). Interestingly, the relative frequency of occurrence of ship tracks within the Californian SECA was found to drop by 73 % (Gryspeerdt et al., 2019b) following this emission control area policy change.

The primary goal of the Atmospheric Composition and Radiative forcing changes due to UN International Ship Emissions regulations (ACRUISE) project is to determine how this international regulation affects aerosols, clouds, and climate. A series of flights as part of the UK ACRUISE project using a wide range of instrumentation on the Facility for Airborne Atmospheric Measurements (FAAM) aircraft were conducted in summer 2019 through the English Channel and off the west coasts of Portugal and the UK. The main goals of the flights, supported by large-eddy simulations and satellite cloud detection, were to quantify ship emission rates and study cloud properties in ship tracks. Analyses to date that compare 2019 observations (Yu et al., 2020) outside and inside of the Sulfur Emission Control Areas (SECA) indicate much lower emissions of sulfur dioxide gas, particulate sulfate, and aerosol particles large and/or hygroscopic enough to act as CCN within the SECA despite higher shipping traffic density (taken to be a proxy for the open ocean after 2020). Post-regulation flights took place during the summer of 2021 primarily off the west coast of France (instead of Portugal as was the case in the 2019 flights) and will be used to verify the anticipated changes in emissions and cloud sensitivities. Long-term ground observations of sulfate aerosol (and sulfur isotopes) at the Penlee Point Atmospheric Observatory in the southwestern UK and at the ARM-ENA site at the Azores is being examined within ACRUISE to quantify the impact of shipping regulation on the aerosol sulfur burden. Finally, both global modeling and satellite cloud detection (aided by machine learning) in conjunction with air mass trajectory analyses will be used to estimate the total radiative effect of ship emissions. In contrast to earlier studies, ACRUISE aims to quantify the impacts of ship emissions not only in the near field (e.g., ship tracks, which only occur for a very small fraction of the time), but also in the far field, where diffuse emissions are expected to affect the background aerosol concentrations. The extent to which the 2020 policy change has influenced the global occurrence of ship tracks or climate at large is a current research question under investigation in ACRUISE and, at least for 2020–2021, may be obscured by COVID-19-related effects on both decreased shipping traffic (March et al., 2021) and enforcement efforts.

Overall, ship emissions provide a useful laboratory to study process-level physics of ACI in ship tracks as well as for quantifying the radiative effects on shallow marine cloud systems more broadly over entire shipping corridors and even the globe. Unique changes in policy and regulations directly influence ship emissions, and these changes are currently creating an interesting experiment to examine, but it may take several years for a clear signal to emerge from the radical emissions changes in 2020 due to the COVID pandemic (see Sect. 2.8.2).

### 2.2 Industrial sources

Industrial aerosol sources are responsible for a large part of the global anthropogenic aerosol forcing (Stevens et al., 2017). This means that cloud responses to emissions originating from a subset of strong industrial sources (e.g., smelters) may serve as an analogue for global anthropogenic impacts (Toll et al., 2019). Industrial perturbations cover a variety of spatial scales (Fig. 3): from isolated factories with a single chimney inducing a narrow ship-track-like perturbation (Rosenfeld, 1999) to continental-scale industrial perturbations (Goren and Rosenfeld, 2015; McCoy et al., 2018). While cloud responses to emissions originating from localized isolated sources provide the highest signal-to-noise ratio and are highly informative for process-level understanding (Toll et al., 2019), analysis of continental-scale perturbations is probably more relevant to global forcing estimates (McCoy et al., 2018). Industrial sources often emit constantly, although emissions can change over time. As an example, copper and nickel production facilities in Norilsk, Russia, emit more than 1 Mt of SO_2_ each year, i.e., more than 1 % of global anthropogenic SO_2_ emissions (Fioletov et al., 2016). Such strong localized emissions induce a high contrast between clouds affected by the emissions of the Norilsk smelters and nearby less polluted clouds (Trofimov et al., 2020). The opening and closing of large factories and implementation of desulfurization devices can lead to rapid changes in emissions (Fioletov et al., 2016), providing additional insight into aerosol impacts on clouds. On the downside, since industrial sources are most often clustered into larger industrial regions, and therefore create a polluted background, it can be difficult to observe the impact of individual sources.

One of the first discussions of the potential for aerosol–cloud–climate interactions in the literature involves the effect of pollution from an industrialized port in southeastern Australia (Twomey, 1974). An early confirmation of the Twomey effect of increasing *N*_d_ from pollution came from flights through plumes emitted by the Centralia coal plant in Washington State (Hobbs et al., 1980). Figure S1 shows sulfur dioxide emissions from the power industry and combustion for manufacturing sectors from EDGAR for 2015. The large concentration of pollution sources in rapidly industrializing regions like southern and eastern Asia is apparent. Given the number of sources in these regions, it may be hard to use individual power plants or industrial sites as opportunistic experiments. In other more remote locations like Australia and Canada, however, there appear to be more frequently isolated but large sources. Toll et al. (2019) studied continental clouds influenced by industrial pollution in Russia, Kazakhstan, Canada, and Australia. Stratiform clouds over land responded to isolated pollution sources in much the same manner as marine stratiform clouds did. An expanded analysis, focusing on the Norilsk pollution hotspot in Russia but including some data from the United States, Europe, and eastern Asia in addition to that used in Toll et al. (2019) confirmed that competing LWP adjustments in varying conditions average out to a small offset of the Twomey effect (Trofimov et al., 2020). It is noteworthy that this result, where the focus is more in continental areas, contrasts with more significant Twomey effect offsets in the shipping lane study of Diamond and Wood (2020).

Like ship emissions, industrial sources provide unique opportunities to study ACI but with the added advantage of having more information with regards to the source and characteristics of the emitted aerosol. While these cloud systems are commonly found over land areas and are less of a direct analog for anthropogenic forcing over the oceans, there is also the potential to have greater coverage of ground-based observations, and a greater range of particle types and background conditions, to aid in quantifying ACI. In addition, industrial sources have fixed locations and often emit continuously, enabling analysis of cloud perturbations for various cloud types and meteorological conditions characteristic to the specific location.

### 2.3 Volcanoes

Large explosive volcanic eruptions have long been studied for their ability to affect the climate by injecting aerosols into the stratosphere, blocking sunlight and causing a temporary cooling (Robock, 2000). It has now been recognized that passive degassing and weakly explosive or effusive eruptions, in which volcanic emissions remain at relatively low altitudes, can also produce a cooling effect via their indirect effects on clouds (Graf et al., 1997; Gassó, 2008; Schmidt et al., 2012). Ship-track-like perturbations have been observed downwind of volcanoes at Hawai’i, South Sandwich Islands, Kuril Islands, and Vanuatu Islands (Gassó, 2008; Yuan et al., 2011; Ebmeier et al., 2014; Toll et al., 2017; Gryspeerdt et al., 2019b; Toll et al., 2019) and also show increased *N*_d_, decreased drop effective radius *R*_e_, increased cloud brightness, and variable effects on LWP in larger-scale eruptions (Seifert et al., 2011; McCoy and Hartmann, 2015; McCoy et al., 2018).

Satellite measurements between 1978 and 2014 estimate an average SO_2_ flux of 23 ± 2 Tg yr^−1^ into the troposphere from passive (non-eruptive) degassing (Andres and Kasgnoc, 1998; Carn et al., 2017). The average SO_2_ emission rate from explosive and effusive eruptions is 3 Tg yr^−1^, of which about 1 Tg yr^−1^ is injected into the upper troposphere and stratosphere (Carn et al., 2016). Modeling studies indicate that passive degassing and weakly explosive or effusive eruptions elevate the tropospheric background level of sulfur and can induce a significant radiative forcing (Schmidt et al., 2012). The Kīlauea volcano on the island of Hawai’i is an effusive volcano that erupted continuously from 1983 to 2018, with large SO_2_ emissions in 2008 and 2018. Kīlauea induces significant perturbations in *R*_e_ downwind of the island of Hawai’i (Yuan et al., 2011; Ebmeier et al., 2014). The eruptions resulted in a 3 standard deviation increase in *N*_d_ in the downstream wake of the plume. Mace and Abernathy (2016) also found higher cloud top heights in the Kīlauea plume relative to adjacent clouds unaffected by the plume. Finally, Kīlauea emits continuous SO_2_ for long periods of time (months) and thus has the advantage of perturbing clouds over a longer timescale and region and may be more relevant (compared to ship tracks, which are shorter lived) to the climate scale (Glassmeier et al., 2021).

The 2014–2015 Holuhraun eruption in Iceland lasted 6 months (31 August 2014 to 28 February 2015) and emitted a total of around 11 Tg of SO_2_ into the lowermost troposphere (Gislason et al., 2015). Daily SO_2_ emission rates averaged 0.06 Tg d^−1^ (Gislason et al., 2015; Schmidt et al., 2015), which dwarfs other such eruptions in recent history. Space-based, multi-angle imaging of the eruption on 11 September 2014 shows sulfate particles growing in size downwind, and at about 350 km from the volcano, at an approximate plume age of 10–12 h, the particles merge into cloud at the same elevation (Flower and Kahn, 2020). These observations offer a constraint on the timescale of downwind particle processing such as aggregation, deposition, and/or new particle formation under different atmospheric static stability, relative humidity, and wind shear conditions at plume altitude, and notably in this case, particle hydration and likely activation.

Another analysis of the 2014–2015 Holuhraun fissure eruption in Iceland revealed that global climate models can represent the decrease in *R*_e_ observed in satellite retrievals. Malavelle et al. (2017) show that the increases in LWP are far from uniform across models (e.g., HadGEM-UKCA averages to a zero LWP adjustment with significant regional increases and decreases, while other models show a wide variation). Gettelman et al. (2015) estimated that emissions from the Holuhraun eruption in Iceland resulted in a regional radiative forcing of −0.21 Wm^−2^, 80 % of which was attributed to ACI. Had this level of emissions occurred in summer rather than in autumn, the radiative forcing would have been much larger (−0.61 Wm^−2^, 94 % of which is attributable to ACI) (Gettelman et al., 2015). During summer the radiative effects are larger due to a greater solar flux and a higher burden of sulfates from gas-phase oxidation.

The last major volcanic eruption globally occurred at Mount Pinatubo in 1991. Satellite and modeling capabilities to observe and model such events have greatly improved since, and a future major eruption would offer a unique natural experiment for further ACI studies. The eruption of Pinatubo and the associated suite of measurements proved a catalyst for improving our knowledge and understanding and modeling of stratospheric aerosol. Even after 25 years, studies into Pinatubo show no sign of abating, indicating the longevity of such important natural analogues to the science community. In much the same way, opportunistic experiments found in large degassing events such as those that occurred in Iceland and Hawaii provide a similarly compelling case study for aerosol–cloud interactions. Volcanoes thus serve as another useful laboratory to study ACI because they can emit significantly more aerosols and SO_2_ than typical ships or industrial plants (see Sect. 4.3), but their episodic nature and uncertain emissions can make interpretation and quantification of ACI relationships challenging.

### 2.4 Fires and biomass burning

Agricultural burning as a promising natural laboratory for studying aerosol–cloud interactions was proposed as early as the 1960s, as there appeared to be a decrease in precipitation following an intensification of burning associated with sugar cane production in northeastern Australia (Warner, 1968; Warner and Twomey, 1967). Biomass burning events around the globe have been recognized as promising targets for studying aerosol–cloud interactions (Kaufman and Nakajima, 1993; Rosenfeld and Lensky, 1998; Haywood et al., 2003) and wildfire-driven thunderstorms, for example, that can manifest as pyrocumulonimbus clouds through intensive and widespread surface burning (Peterson et al., 2017; Lee et al., 2020; Y. Zhang et al., 2019b).

Biomass burning can emit black carbon into the atmosphere and influence cloud properties in a myriad of ways. Black carbon can strengthen the effective radiative forcing by aerosol–cloud interactions by reducing entrainment when it resides above the cloud but burn off the cloud when it resides in the cloud layer (Johnson et al., 2004). Furthermore, methods that relate cloud properties to above-cloud rather than below-cloud aerosol concentrations likely misrepresent aerosol microphysical effects on clouds (Diamond et al., 2018). At high smoke concentrations, clouds move from an aerosol-limited to an updraft-limited regime in which cloud sensitivity to further aerosol increases is limited (Kacarab et al., 2020). Meanwhile some of the lowest aerosol concentrations observed at Ascension Island (farther from the source of the biomass burning aerosols) are likely due to incloud scavenging (Pennypacker et al., 2020). Surprisingly, smoke from subequatorial Africa influences clouds north of the Equator in southern West Africa as well (Haslett et al., 2019). In addition, Wang et al. (2018) revealed contrasting responses of lightning to aerosol optical depth (AOD) for smoke and dust aerosols in Africa. Lightning frequency increases with AOD (for AOD < 0.3) but then decreases for dust and remains flat for smoke with further AOD increase. However, this result does not imply causality, and meteorological co-variability may confound the AOD–lightning relationship (Wang et al., 2018).

**Figure 5.**
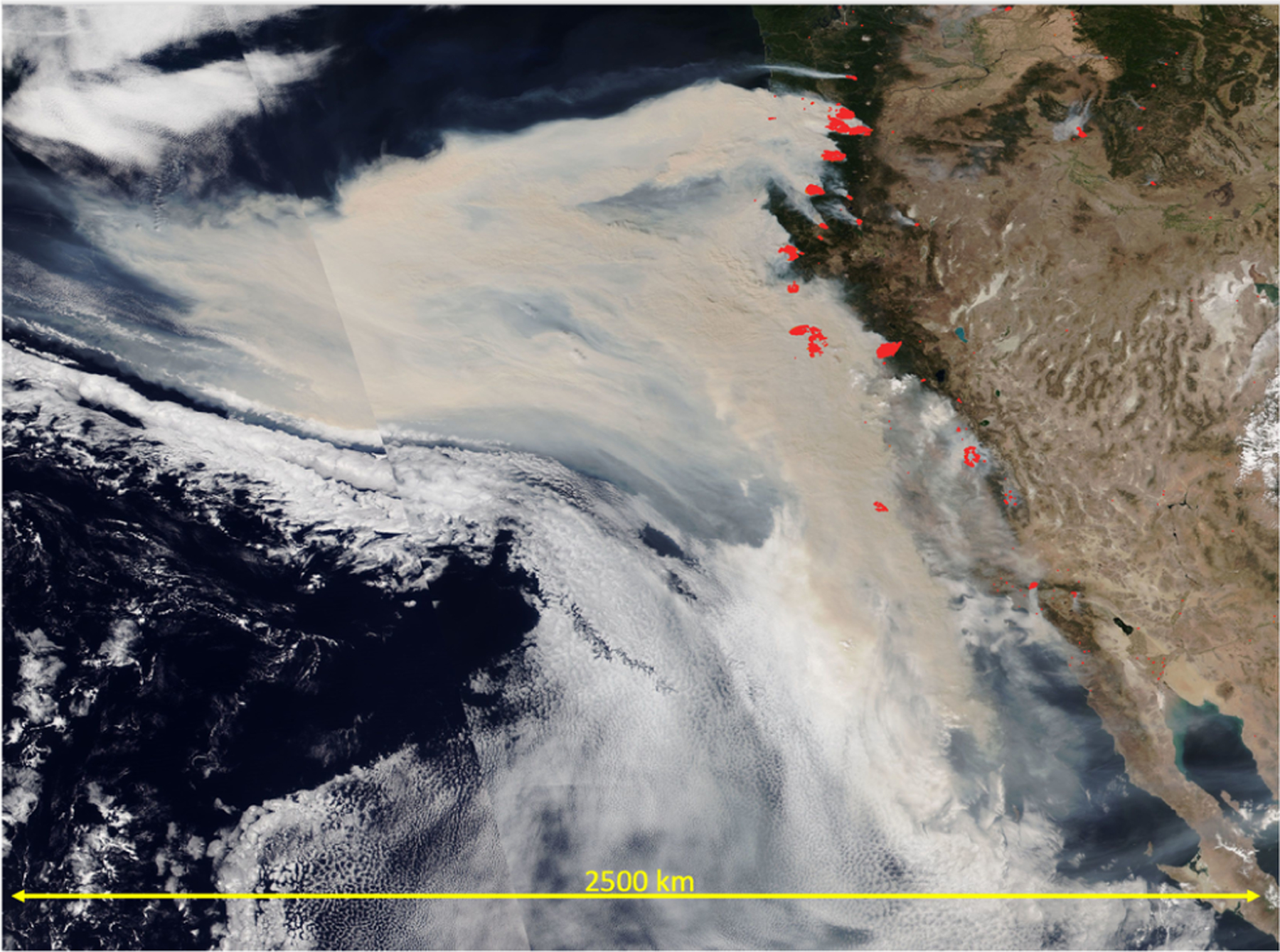
Smoke plume and clouds at the US west coast on 9 September 2020 as seen by NOAA-20 Visible Infrared Imaging Radiometer Suite.

Recent fire seasons in California in 2020 and in Australia in 2019/20 generated many large-scale smoke plumes (example in Fig. 5). These strong fire seasons have the potential to induce large-scale anomalies in cloud properties. The NiCE campaign and subsequently the 2016 Fog and Stratocumulus Evolution Experiment (FASE) included numerous flights and quantified the impacts of biomass burning plumes on stratocumulus clouds including both when the plumes were above (Mardi et al., 2018) and in/below clouds (Brioude et al., 2009; Mardi et al., 2019). Analysis of cloud anomalies compared to long-term climatology is challenging in the case of fires, as it is difficult to separate the aerosol effect from the influence of weather anomalies that favor the occurrence of the extreme fire season in the first place.

Some individual wildfire plumes were analyzed in the studies of Toll et al. (2019) and Trofimov et al. (2020). Another opportunistic experiment is the smoke–cloud system that develops seasonally over the southeast Atlantic stratocumulus deck (Zuidema et al., 2016, 2018), where it is obvious that the smoke can be traced to the effects of agricultural burning over the continent rather than processes occurring over the ocean. The regional-scale perturbation lasts in some form for 4 or 5 months each year. The aerosol contribution from the smoke clearly overwhelms other aerosol sources in the free troposphere and on occasion dominates the marine boundary layer aerosol population. However, meteorological influences (e.g., the atmosphere stability profile) still play a major role in any observed cloud properties (Wilcox, 2010; Adebiyi et al., 2015). Finally, the large vertical and horizontal extents of smoke plumes make disentangling aerosol radiative effects caused by enhanced solar absorption over both the continent and ocean a challenge.

### 2.5 Hemispheric differences

**Figure 6.**
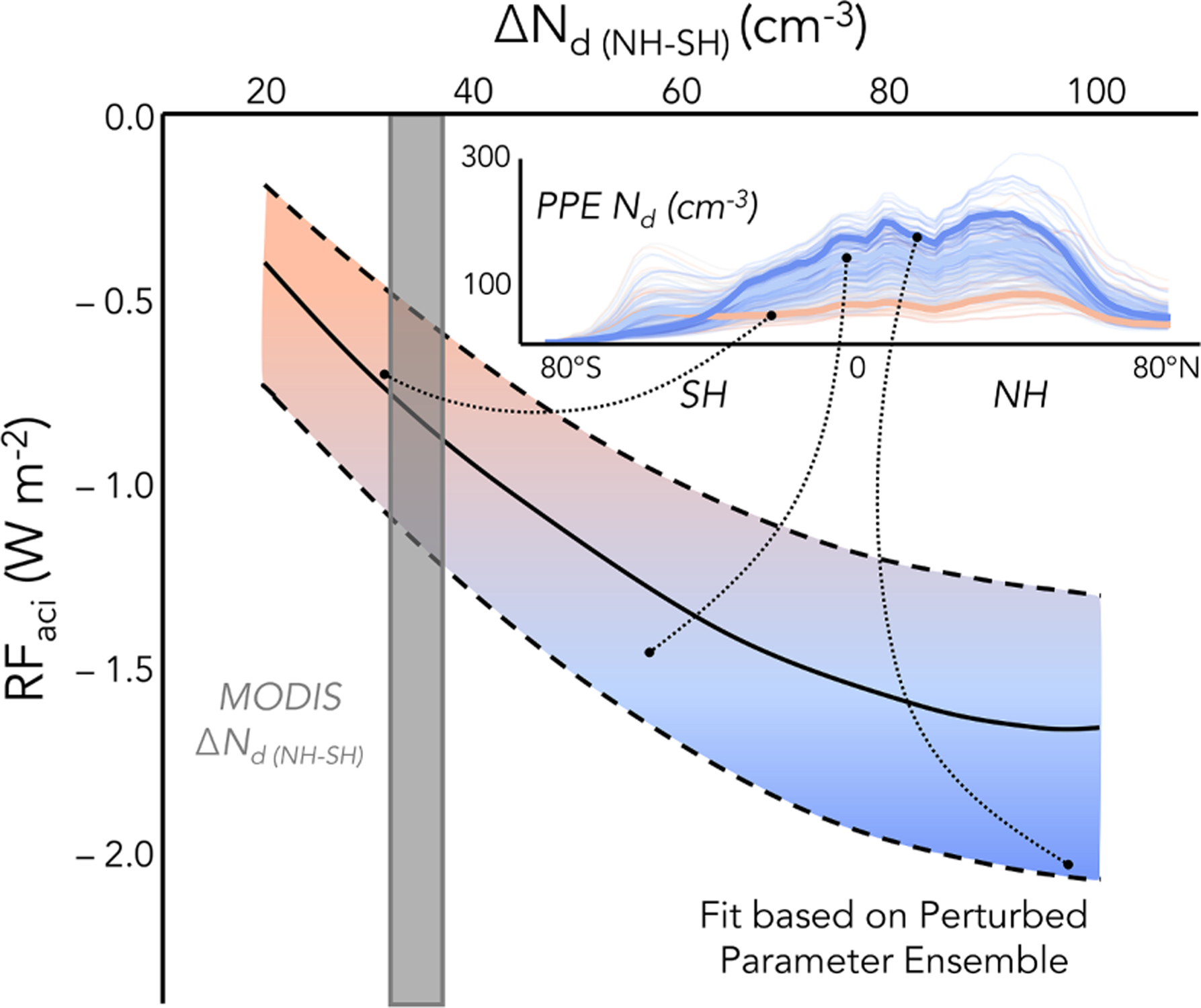
Example of using the hemispheric contrast in *N*_d_ (Δ*N*_d (NH-SH)_) to constrain radiative forcing associated with aerosol–cloud interactions (RF_aci_). A smaller hemispheric *N*_d_ contrast has a smaller RF_aci_ magnitude and thus less cooling. Curves (solid line is linear fit, dashed lines are 95 % prediction bands) are based on perturbed parameter ensemble (PPE) results described in McCoy et al. (2020) and approximately shaded by RF_aci_ (blue for more aerosol cooling, orange for less). The inset shows zonal *N*_d_ of individual PPE members colored by RF_aci_ approximately corresponding to shading along the best-fit line (dotted lines). The gray bar shows 95 % confidence on the inter-annual range of Δ*N*_d (NH-SH)_ from MODIS satellite estimates between 2003–2015 (Grosvenor and Wood, 2018), yielding an observational constraint on RF_aci_ between −1.2 and −0.6 Wm^−2^ (McCoy et al., 2020).

The Southern Hemisphere (SH), in particular the remote Southern Ocean (SO), is thought to be our closest present-day (PD) analog to the pre-industrial (PI) aerosol state (Schwartz, 1988; Hamilton et al., 2014). Hemispheric differences in aerosols and clouds may thus provide a potential natural laboratory. *R*_e_ is smaller in the Northern Hemisphere (NH) (Han et al., 1994; Feng and Ramanathan, 2010), and *N*_d_ is larger (Feng and Ramanathan, 2010; McCoy et al., 2020) compared to the SH. However, high values of *N*_d_ can be found in pristine conditions over the ocean when clouds are coupled to a surface under conditions of high wind (McFarquhar et al., 2020). The hemispheric contrast between cloud properties in the more pristine SH and the more polluted NH is a unique form of natural laboratory for estimating the bulk effect of natural and anthropogenic aerosol emissions on our climate. Several studies have employed this method to understand the PI environment, estimate the change in climate due to industrialization, and improve the accuracy of our future climate predictions by constraining radiative forcing by aerosol–cloud interactions (RFaci) and thus reducing uncertainty in effective radiative forcing by aerosol–cloud interactions (ERFaci) (Bellouin et al., 2020). Boucher and Lohmann (1995) used the hemispheric difference in *R*_e_ to evaluate the robustness of RFaci simulated in several global climate models (GCMs) after prescribing a relationship between sulfate mass and *N*_d_. Feng and Ramanathan (2010) found that a chemical transport model driven by reanalysis meteorology was able to produce a difference in *N*_d_ between the NH and SH that is consistent with hemispheric contrasts in satellite retrievals of *R*_e_ and cloud optical depth. When comparing to satellite studies, McCoy et al. (2020) found that the hemispheric *N*_d_ contrast is overestimated by a collection of CMIP5 (Ghan et al., 2016) and development GCM simulations (Mulcahy et al., 2018), as well as a perturbed parameter ensemble (PPE) exploring parametric uncertainty (Yoshioka et al., 2019). This bias was shown to be a result of models producing uniformly too little SH *N*_d_, and thus too little inferred PI *N*_d_, while also producing increasingly too much NH *N*_d_ with increasing RFaci. Application of the *N*_d_ contrast to the PPE was able to constrain RFaci by eliminating overly negative RFaci values (see example in Fig. 6), producing an RFaci range consistent with independent analysis methods (e.g., Bellouin et al., 2020) and further substantiating the usefulness of the hemispheric contrast methodology.

### 2.6 Long-term trends

Long-term trends in aerosol driven by economic growth and/or policy-driven reductions in pollution may also arguably serve as natural laboratories with the benefit that the long timescales minimize the effect of weather noise on results. For instance, the decrease in cloud reflectance between the 1980s and 1990s has been called the “Gorbachev” effect as it is related to the economic restructuring of eastern Europe following political changes that caused decreased emissions of aerosols and their precursors (Krüger and Graßl, 2002). The co-incident upward trend in surface solar radiation (Wild et al., 2005) caused by both ACI and clear-sky aerosol radiative interactions (ARIs) was found useful as an emergent constraint on simulated total aerosol effective radiative forcing (ERF) in the CMIP5 multi-model ensemble (Cherian et al., 2014). In other regions, there are large discrepancies between surface radiation trends and model results (Stjern et al., 2011; Moseid et al., 2020).

**Figure 7.**
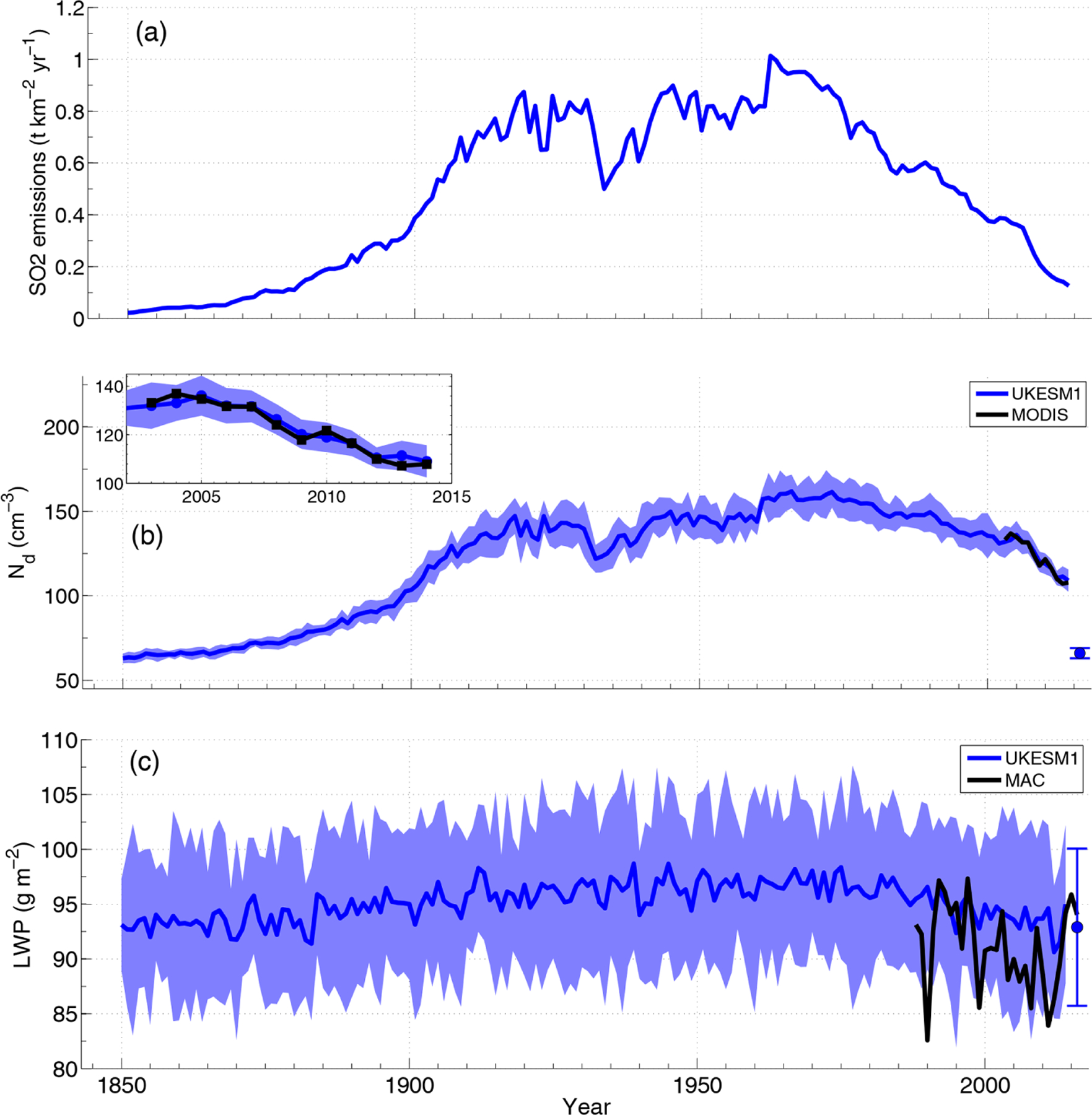
Time series of the annual mean CMIP6 emission rate for anthropogenic SO_2_ **(a)** for the continental US region (26–50° N, 70–100° W; land-only grid points), the cloud droplet number concentration (*N*_d_, **b**), and the all-sky (i.e., including both cloudy and clear parts of grid boxes) liquid water path (LWP, **c**) for a region in the Atlantic Ocean downwind of the US (26–42° N, 56–80° W; ocean-only grid points). Lines are shown for the UKESM1 model ensemble mean, the MODIS satellite instrument using the collection 5.1 product, and the MAC microwave satellite LWP dataset. The blue shading denotes ±2 times the intermodel standard deviation across the ensemble. The error bar plotted at the year 2016 for the *N*_d_ and LWP plots shows the ±2*σ* range of the annual average values from the pre-industrial control run along with the time mean (blue dot). The inset figure in the *N*_d_ plot shows a closeup of the time period for which observations are available using the same axes.

Figure 7 shows that, according to the CMIP6 emissions database (Mulcahy et al., 2020), aerosol-generating SO_2_ emissions from the continental US increased steadily from 1850 to around 1910, when they stabilized and then later dropped fairly rapidly from just after 1960 until the end of the record in 2014. The latter decrease is associated with the various federal Clean Air and Air Pollution acts, the first of which was introduced in 1955, and is also supported by OMI observations of atmospheric SO_2_ concentrations (McCoy et al., 2018) for the period after 2003. The SO_2_ emission changes are mirrored by *N*_d_ changes in the ensemble mean CMIP6 UK Earth System climate model (UKESM1; Sellar et al., 2019) for a region in the North Atlantic that is downwind of the US. The model *N*_d_ and trend match those from MODIS very well over the 2003–2014 period, giving confidence in the CMIP6 emissions and the ability of this model to accurately translate emissions into changes in cloud properties, which involves several stages. However, Robson et al. (2020) and Grosvenor and Carslaw (2020) show that this model does exhibit biases in *N*_d_ and its trends in other regions.

It is tempting to relate these changes in *N*_d_ to observed and simulated trends in cloud fraction, LWP, and shortwave fluxes. For example, Robson et al. (2020) suggest that the negative upwelling shortwave top-of-atmosphere flux trend in UKESM1 for the wider North Atlantic region is too strong compared to CERES, with the model also displaying a positive bias in upwelling shortwave top of atmosphere fluxes coincident with a cloud fraction that is too high compared to CALIPSO (see also Grosvenor and Carslaw, 2020). The overly strong trend may be interpreted as an overly strong cloud response to aerosol. However, natural multi-decadal variations in the sea surface temperature in the North Atlantic (which are not necessarily captured by models) could also lead to cloud trends unrelated to aerosols (Vaideanu et al., 2018). Figure 7 provides a demonstration of this through the time series of the all-sky LWP (i.e., including the zero LWP values in the clear parts of grid boxes and hence showing the combined effect of both cloud thickness and cloud area fraction changes) from the CMIP6 UKESM1 model for the same region downwind of the US where large negative *N*_d_ trends over the 1960–2014 period were described above. A negative 1971–2014 LWP trend of −0.1±0.03 gm^−2^ yr^−1^ (significant to > 99.9 %) is apparent in the mean of the 16-member ensemble. However, the magnitude of the LWP change over this period is much smaller than the inter-ensemble spread in LWP for a given year (shading), and there is a large range of trends across the ensemble when computed using individual members (−0.21 to −0.02 gm^−2^ yr^−1^). This implies that, to the extent that we can trust the model, for the same forcing a wide range of trends is equally plausible due to natural variability and that it would therefore be difficult to attribute an observed trend to a forcing (e.g., the aerosol forcing). This is supported by the observed LWP time series from the MAC (Multisensor Advanced Climatology) microwave satellite LWP dataset (Elsaesser et al., 2017); however the dataset is also very noisy, and the 1988–2014 trend is not statistically significant.

Furthermore, climate models predict that greenhouse-gas-driven cloud changes (and by extension temperature-driven changes, i.e., cloud feedbacks) are very likely to have occurred over the historical period in addition to aerosol-driven changes and natural variations (Norris et al., 2016; Cherian and Quaas, 2020; Schneider, 2020). Thus, any observed cloud changes include natural variability, aerosol–cloud interactions, cloud feedbacks (due to surface temperature change), and cloud adjustments to the forcing (CO_2_, aerosols, etc.) evolution. This makes it difficult to infer cloud–aerosol adjustments from long-term trends since it requires knowledge of the non-aerosol-driven changes. The agreement over the satellite era between the modeled CMIP5 cloud fraction trends and those from observations as demonstrated in Norris et al. (2016) gives some confidence in the ability of the models to represent changes in clouds in response to the different balance of forcings, but the uncertainty does not allow an easy quantification of the forcing. Further uncertainty comes from the possibility that spurious observed trends can be introduced due to several issues in satellite data such as instrument and platform changes, orbital drift, calibration issues, and other unidentified stability problems, in addition to differences in retrieval algorithms (Evan et al., 2007; Levy et al., 2013; Norris and Evan, 2015; Norris et al., 2016).

Rapid changes in anthropogenic emissions have occurred over east and south Asia (especially China) over the last few decades. China’s aerosol loading increased most strongly during the rapid industrial growth of the 1970s to 1990s, followed by gentle increases from 2000–2010 and finally a decrease thereafter as a result of increased political attention and action on air pollution (Jin et al., 2016; Q. Zhang et al., 2019a). Accompanying these trends were changes in surface radiation, temperature, and precipitation, some of which were attributed to the influences of ARI and ACI, at least to some extent (Li et al., 2016; Z. Li et al., 2019b; Shi and Brasseur, 2020). Yet, different types of aerosols were identified to play rather different roles, which helps explain the opposite decadal trends in severe thunderstorms in central China (where absorbing aerosols dominate) and southeast China (where hygroscopic aerosols dominate; Yang et al., 2013; Yang and Li, 2014).

Increases in *N*_d_ over the East China Sea were observed from the 1980s to the 2000s (Bennartz et al., 2011). Coincident with this is a decreasing trend in cloud fraction in the same region (Xia, 2010; Norris et al., 2016), which may hint at a reduction in cloudiness with increasing *N*_d_ and decreased surface incoming solar radiation, although the trend could also be due to other drivers. McCoy et al. (2018) observed a stabilization of *N*_d_ over China in the 2000s followed by a decreasing trend in the 2010s. In this more recent period (2006–2015), Benas et al. (2020) document an increase in LWP and cloud fraction that, if caused by the decrease in aerosol, would imply a reduction in both quantities with increasing aerosol.

More generally, Cherian and Quaas (2020) demonstrated that in the CMIP6 multi-model ensemble, aerosol optical depth (AOD) and *N*_d_ trends compared favorably to trends derived from MODIS over four different regions with different behaviors of anthropogenic aerosol sources. In contrast, CMIP5 model trends were erroneous, e.g., over northwestern North America, but also over China. Both CMIP5 and CMIP6 models generally showed trends in LWP and cloud fraction that were inconsistent with the pattern derived from MODIS, although the observed trends were rarely statistically significant.

A MODIS analysis examining negative long-term AOD and aerosol index, possibly a better measure of finer-mode aerosol (Nakajima et al., 2001) and hence possibly CCN (Stier, 2016), found that *N*_d_ also decreased while LWP was relatively unaffected in 15 years of MODIS data off the eastern coasts of the United States and China and western coast of Europe (Bai et al., 2020). This is in line with the other opportunistic experiments that also indicated small LWP adjustments. However, as discussed above, extreme caution is required when interpreting trends in cloud properties as being caused by aerosol forcing even when there are strong concurrent aerosol trends. Ways forward may involve using climate models or machine learning to identify situations when cloud trends are likely to be caused by aerosol rather than other factors and focusing on those for the quantification of cloud–aerosol adjustments. Other approaches include stratifying vast amounts of satellite data into small bins in meteorological variables and examining aerosol–cloud relationships within bins to control for co-varying meteorology (e.g., Zamora and Kahn, 2020).

Overall, long-term trends are useful for correlating observed changes in clouds and radiative effects to aerosols but are likely not suited for process understanding of ACI unless new analysis techniques can overcome the abovementioned issues.

### 2.7 Weekly cycle

**Figure 8.**
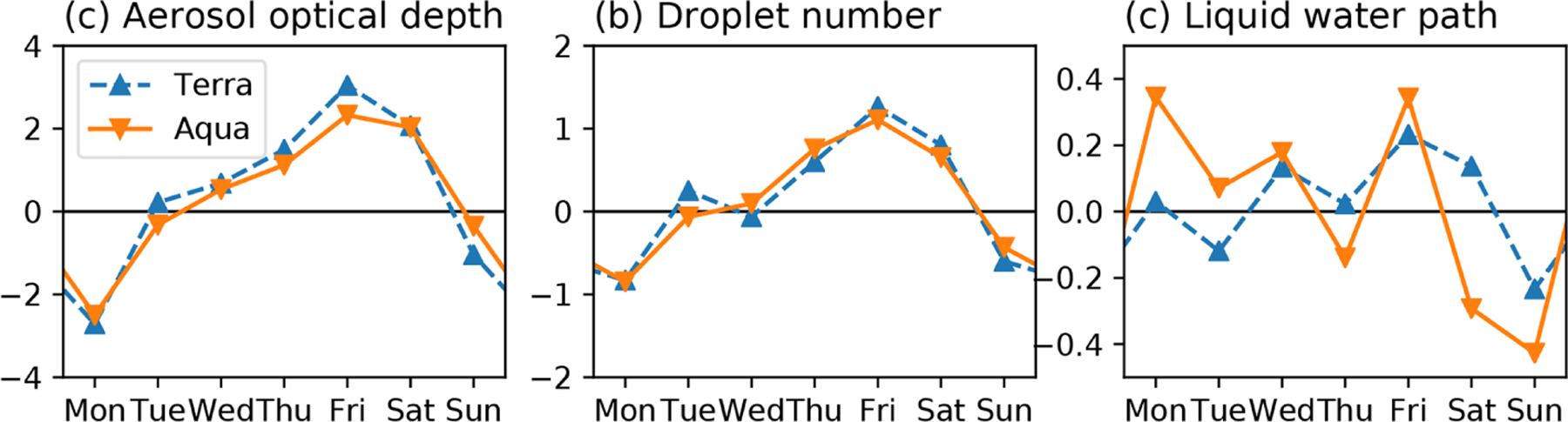
Weekly cycle of **(a)** AOD, **(b)** *N*_d_, and **(c)** LWP, in percent deviation from the temporal average, as an average over continental Europe (35 to 70° N, 10° W to 30° E, land only) from MODIS Collection 6 retrievals (Levy et al., 2013; Platnick et al., 2017), where *N*_d_ and LWP are computed assuming adiabatic clouds (Grosvenor et al., 2018). In an update to Quaas et al. (2009), the period from 2003 to 2020 is used for Terra (10:30 LT, upward-pointing blue triangles, dashed line) and Aqua (13:30 LT, downward-pointing orange triangles, plain line).

A 7 d cycle is not a common naturally occurring phenomenon, and the regional variation in weekdays with maxima and minima in anthropogenic reactive gases offers clear evidence of an anthropogenic signal (Beirle et al., 2003). Weekend effects have been directly tied to the study of ACI in particular. Weekend declines and weekday peaks in pollution have also been observed in satellite *N*_d_ and reconstructed in climate models in Europe (Quaas et al., 2009). There is a clear weekly cycle in AOD with minima on Mondays and a co-incident cycle in *N*_d_ (Fig. 8). However, trends in any other quantity (including LWP) are unclear or ambiguous. Higher weekday aerosol levels in the United States have been argued (controversially) to be linked to the invigoration of storms (Schultz et al., 2007; Bell et al., 2008, 2009; Rosenfeld and Bell, 2011). Similarly, lower weekend levels of absorbing aerosol have been hypothesized to suppress thunderstorm activity in central China whereas higher weekday levels of more hygroscopic aerosol in southeast China have been hypothesized to invigorate storms in that region (Yang et al., 2016). However, the occurrence of a single maximum and minimum each, among just seven instances, is rather likely, so that attribution using model evidence is required to corroborate conclusions (Barmet et al., 2009; Quaas et al., 2009; Stjern, 2011; Daniel et al., 2012; Sanchez-Lorenzo et al., 2012). The 7 d cycles in geophysical quantities do not typically arise by natural variability, and if they can be identified with certainty this laboratory may provide a clear pathway to attributing an aerosol influence on clouds.

### 2.8 Particular events

Effects on aerosols from short-term events at the regional or global scale may also provide a natural laboratory if the perturbations are large or abrupt enough. These events range in scale from a single holiday to sudden global economic changes (see below). Recurring holidays and days of rest have been investigated around the world (Forster and Solomon, 2003; Sanchez-Lorenzo et al., 2012; Earl et al., 2015; Pereira et al., 2015). Traffic and firework effects have sizable impacts on gaseous and particulate pollutant concentrations during the extended Chinese Lunar New Year celebrations (Tan et al., 2009).

#### 2.8.1 Emission events in China

**Figure 9.**
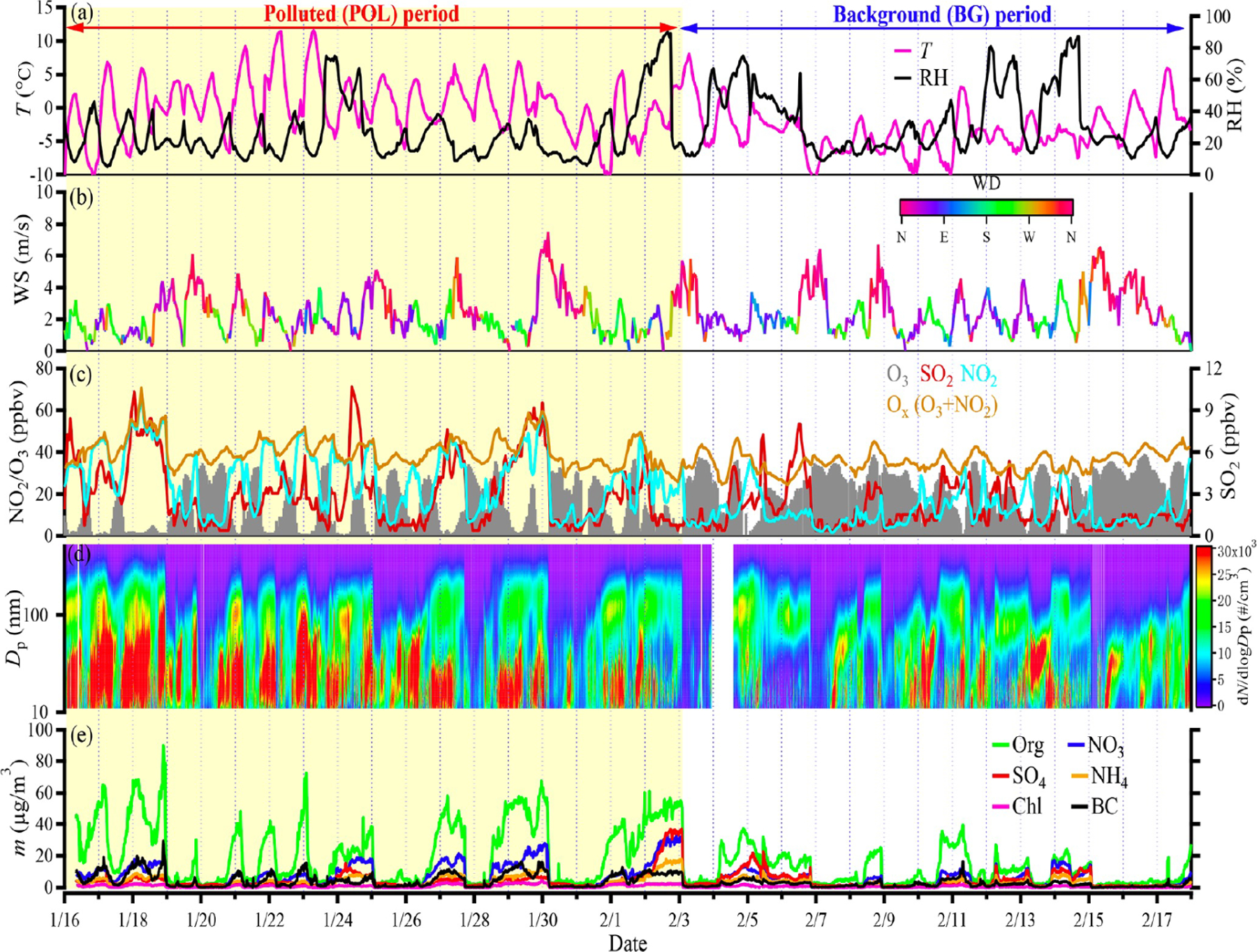
Time series of meteorology and aerosol precursor gases and species before (polluted) and during (background) the 2019 Chinese Spring Festival (16 January to 17 February 2019) in Beijing, China. **(a)** Ambient temperature (*T*) and relative humidity (RH), **(b)** wind direction (WD) and speed (WS), **(c)** volume mixing ratios of trace gases [O_3_, SO_2_, NO_2_, and O_*x*_ (O_3_ + NO_2_)], **(d)** the aerosol particle number size distribution measured by the SMPS, and **(e)** mass concentrations of aerosol chemical species in PM_2.5_ measured by the ACSM and the AE-33. (adapted from Wang et al., 2021a).

There have been a number of special events held in China during which air quality experienced drastic changes over relatively short periods, such as the 2008 Olympics and Paralympics in Beijing (Cermak and Knutti, 2009; Witte et al., 2009; Wang et al., 2010; Guo et al., 2013), 2010 World Expo in Shanghai (Hao et al., 2011), 2014 Youth Olympic Games in Nanjing (Ding et al., 2015), 2014 Asia-Pacific Economic Cooperation meeting (Sun et al., 2016), 2015 China Victory Day parade (Wang et al., 2017; Zhao et al., 2017), and 2016 G20 Summit (K. Li et al., 2019). These have provided unique opportunities to investigate the impact of human activities on air quality, weather, and climate. Perhaps the most famous example of an abrupt, ephemeral change in the environment clearly associated with human decisions is the massive effort to reduce air pollution surrounding the 2008 Beijing Olympic Games. Cermak and Knutti (2009) used a neural network to account for potential meteorological confounders of an aerosol effect from the Olympics-related cleanup. Although they were able to detect a decrease in satellite-retrieved aerosol loading around Beijing during the Summer Olympics, its magnitude was relatively small compared to meteorological variability. Cloud-seeding efforts using silver iodide were carried out ahead of the 2008 Olympics opening ceremony in an attempt to create a downpour but keep the stadium dry, although the efficacy of weather modification above natural variability remains difficult to verify (Flossmann et al., 2019). The annual Chinese New Year Spring Festival holiday is another major, yet more regular, occasion when the vast majority of the population stops working for 2 to 4 weeks, as hundreds of millions of migrant workers return to their hometowns in the countryside. This event results in localized changes to anthropogenic emissions, gaseous pollutants, and fine particulate matter (PM_2.5_) (Tan et al., 2009; Wang et al., 2017). As shown recently by Wang et al. (2021b), sharp reductions were observed during the 2019 festival in virtually all precursor gases (e.g., SO_2_, NO_2_), except ozone (O_3_), and aerosol particle (organic, sulfate, nitrate, BC, etc.) number and mass concentration at all sizes, while the meteorology remained relatively stable prior to and during the festival (Fig. 9). However, even small changes in meteorology can have large implications for cloud radiative properties (Gryspeerdt et al., 2016). There are relatively few studies concerning the impact of these events on meteorological variability (K. Li et al., 2019), partially due to the short periods and thus limited data samples.

#### 2.8.2 COVID pandemic

The global COVID-19 pandemic that emerged and spread around the world in early 2020 created unprecedented socioeconomic changes. The resulting changes in economic activity have been linked with sharp and sudden declines in certain forms of air pollution such as nitrogen oxides in China, Europe, South Korea, and the United States (Bauwens et al., 2020; Liu et al., 2020). However, the effects of the shutdowns on other pollutants like ozone and aerosol particles have proven to be less straightforward (Chang et al., 2020; Diamond and Wood, 2020; Huang et al., 2020; Le et al., 2020; Shi and Brasseur, 2020; Wang et al., 2020; Hammer et al., 2021). Carbon dioxide emissions declined modestly due to shutdown measures worldwide (IEA, 2020; Le Quéré et al., 2020). Strong declines in NO_2_ have been observed in locations including eastern Asia, Europe, the Indian subcontinent, and North America (Bauwens et al., 2020; Diamond and Wood, 2020; Liu et al., 2020). Estimates of changes in PM_2.5_ from ground stations in China range from no or small changes (Silver et al., 2020) to reductions of a third to half (Shi and Brasseur, 2020); follow-up work that accounted for long-term trends by Xian et al. (2021) showed that PM_2.5_ was decreased by 17.13 μm.

Diamond and Wood (2020) found a substantial decline in NO_2_ over China during the February 2020 shutdowns but no clear changes in AOD or *R*_e_ and thus suggested that the February 2020 shutdown effect on regional climate was negligible. In China, the reduction in emissions during the pandemic may have been offset by the shallowing of the planetary boundary layer (PBL) caused primarily by anomalous meteorology (Su et al., 2020). As a consequence, the occurrence of a very serious widespread pollution episode in the midst of the pandemic due largely to the accumulation of pollutants in the shallow PBL posed a special challenge to the evaluation of the influences of the pandemic-related reductions. Loeb et al. (2021) used a multivariate linear regression method to estimate that there would have been a substantial reduction in AOD and aerosol direct radiative effect over China had February and March 2020 not been as humid as they were. However, Andersen et al. (2021), using a gradient boosted regression tree machine learning method, did not find an unequivocal AOD decrease even after controlling for daily meteorology.

Ensembles of simulations with two different Earth system models using emissions reductions from mobility data (Forster et al., 2020) to represent the COVID-19 response show a robust decrease in AOD and increase in *R*_e_ over China in February 2020 but at a level likely too small for observational methods to detect (Gettelman et al., 2021b) due to substantial natural variability in clouds as noted above. Nonetheless, the two models produce a sizable global mean forcing from reduced aerosol–radiation and aerosol–cloud interactions (up to +0.3 Wm^−2^ in May 2020), although much of that effect is from later shutdown measures outside of China and would probably not be distinguishable from noise in actual observations. An assessment of the relative magnitude of the effects of pandemic-induced changes to greenhouse gases, air pollution, and aerosols with a climate model emulator found that ERFaci dominates the response relative to greenhouse gas and ozone changes. The net radiative effect is projected to be a negligible global warming for the next 2 years, followed by slight relative cooling from lowered CO_2_ emissions (Forster et al., 2020; Gettelman et al., 2021b). Unless a prolonged global depression develops in response to the economic shock of COVID-19 and the curtailment of many normal business activities, it seems unlikely that large climate-relevant effects will be detectable in observations (Ming et al., 2021; Fyfe et al., 2021; Jones et al., 2021).

The aviation sector saw some of the most significant changes during the COVID-19 pandemic, with reductions in air traffic of up to 70 % (Gettelman et al., 2021a). Schumann et al. (2021), Gettelman et al. (2021a), and Quaas et al. (2021) have used these changes to try to quantify radiative forcing from contrail reductions. Contrails are linear cirrus clouds formed from aviation exhaust in ice supersaturated regions in the upper troposphere, and like other cirrus clouds, their net affect is to warm the planet (positive ERF). Schumann et al. (2021) found improved correspondence in radiative fields between observations and simulations when contrails were included, and Quaas et al. (2021) found corresponding changes in cirrus at a global scale in regions with large changes in air traffic, implying a global radiative forcing of 0.061 Wm^−2^. Gettelman et al. (2021a) found similar sign changes using a global model, which has an overall contrail radiative forcing from all aviation in 2020 of 0.050 Wm^−2^, helping to validate results of recent assessments of aviation radiative impacts (Lee et al., 2021).

Overall, particular events like the experiments discussed here provide potential opportunities to quantify ACI processes and response to changes in the patterns of anthropogenic emissions. They work best if the emissions changes are known and if sufficient observations are available before and/or after the event to establish a good baseline. One challenge with particular events, especially one-off events that are not repeated, is that meteorological effects may be difficult to disentangle from emissions-related changes and cannot be mitigated by averaging over multiple realizations (as can be done with repeating holidays or within a climate model ensemble). Moreover, care must be taken in selecting a baseline for comparison, as other factors such as long-term policy-driven emission trends or unrelated holiday or weekday effects may have influenced the “no event” counterfactual. Nonetheless, because the emissions perturbations are independent of meteorology and reasonably knowable, these events still hold promise for improving our understanding of causality in aerosol–cloud interactions as long as meteorological and other source variability can be addressed.

## 3 Databases for experiments of opportunity

Over the decades, a growing number of databases have contributed to the increasing knowledge on this topic. Table S2 lists several cited databases that are either publicly available or downloadable through private means. Many of these databases are tagged to specific peer-review publications. Carn et al. (2017) catalog the emission rates of SO_2_ from several hundred passive degassing volcanoes using a combination of satellite retrievals and ground-based measurements. In addition, opportunistic experiments resulting from prominent industrial sites such as Norilsk (Fioletov et al., 2016), persistent and weakly explosive volcanic eruptions, (e.g., South Sandwich Islands’ volcanoes and Ambrym), and significant fire “outbreak” seasons have been logged from satellite imagery in Toll et al. (2019) and Trofimov et al. (2020). Ship track databases identified from MODIS satellite imagery are available for the tracks: (a) off the California coast during summer months of 2002–2004 (Coakley and Walsh, 2002; Segrin et al., 2007; Christensen et al., 2009); (b) off the California, Chilean, and Namibian coasts from 2007–2010 collocated to CloudSat and CALIPSO (Christensen and Stephens, 2011, 2012; Chen et al., 2012; Christensen et al., 2014); (c) off the California coast for studying recent shipping emission regulations (Gryspeerdt et al., 2019b); and (d) globally through the use of machine learning (Yuan et al., 2019).

**Figure 10.**
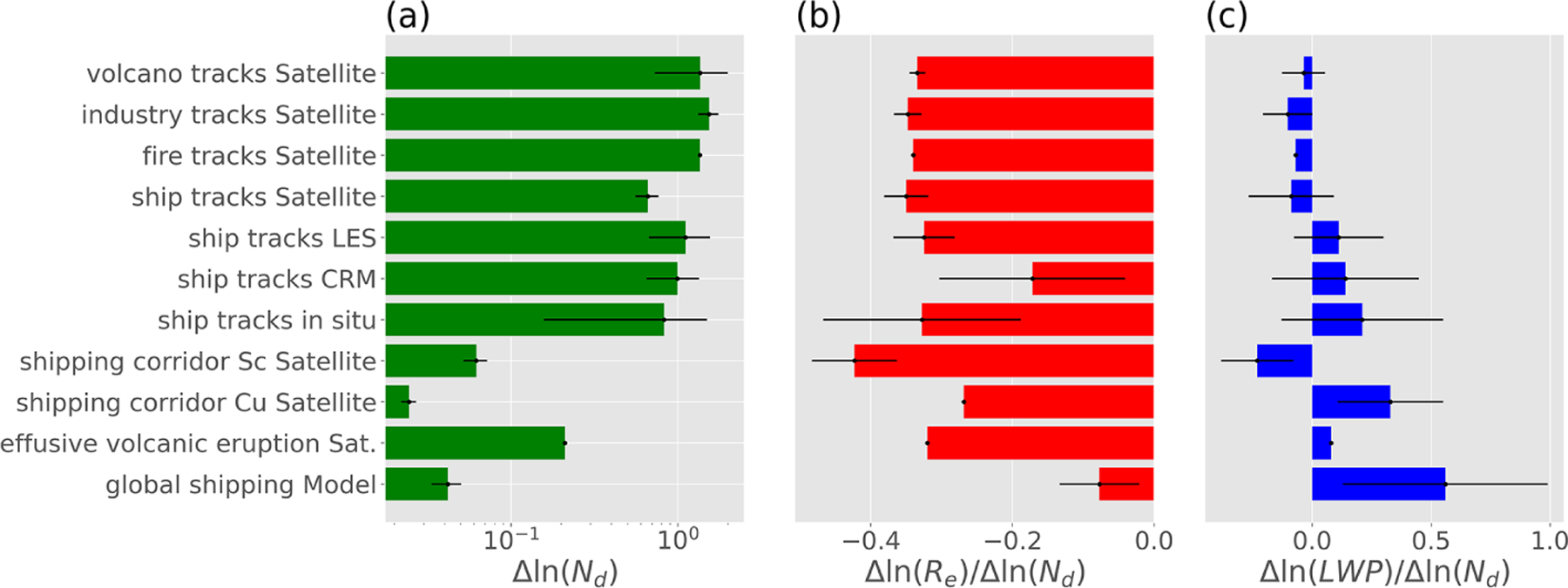
Change in **(a)** cloud droplet concentration Δ*N*_d_, **(b)** sensitivity of cloud droplet effective radius change to *N*_d_, and **(c)** sensitivity of liquid water path change (LWP) to *N*_d_ averaged over numerous studies involving experiments of opportunity. The number of studies going into each category are as follows: volcano tracks Satellite (6), industry tracks Satellite (9), fire tracks Satellite (1), ship tracks Satellite (9), ship tracks LES (6), ship tracks CRM (4), ship tracks in situ (18), shipping corridor Sc Satellite (2), shipping corridor Cu Satellite (2), effusive volcanic eruption Sat. (1), and global shipping Model (3). For a complete listing see Table S1. Error bars represent 1 standard deviation of reported values for each category representing diversity of the mean amongst studies.

These databases and emission estimates have already facilitated fruitful intercomparisons of observations and models (e.g., for GCMs see AeroCom ACI experiment, and for LES intercomparison see Glassmeier et al., 2021), with the synthesized values used to construct the statistics in Figs. 10 and S4 (as described further in the results section). These figures were constructed from published estimates of *N*_d_, *R*_e_, and LWP for numerous opportunistic experiments derived from satellite and in situ observations, large-eddy simulations, cloud resolving, and global model simulations. Figure 10 contains satellite retrievals of volcano, industry, and fire tracks (mostly from Toll et al., 2019) and ship tracks. Shipping corridor perturbation results are from Diamond et al. (2020), effusive volcanic eruption is from Malavelle et al. (2017), and the global shipping model is from Lauer et al. (2007) and Peters et al. (2013). LES (Wang and Feingold, 2009; Berner et al., 2015) and cloud resolving model (Possner et al., 2015, 2018) simulation results for ship tracks are also included in Fig. 10. An exact breakdown of each study used in the figure is provided in Table S1. This list is weighted more to observational studies partly due to their high occurrence in the literature. Thus, publications were sorted by the type of opportunistic experiment and data used in order to provide a comprehensive reference for the expected cloud responses. The expansion and synergistic use of these databases are key to providing constraints on aerosol radiative forcing and cloud perturbations in atmospheric modeling. Finally, while some sources like volcanoes or industrial sites are well documented from public sources, some key data like ship movements are proprietary and unavailable for most researchers.

## 4 Controlling factors

This section lays out prominent “experimental conditions” that studies typically endeavor to hold fixed in a natural laboratory as a means to compare different opportunistic experiments and systematic frameworks to one another. We have compiled a list of peer-reviewed articles that quantify cloud properties and their responses in many opportunistic experiments. An opportunistic experiment means an aerosol perturbation that affects the radiative properties of a cloud scene as a result of a chain of processes: After emission there is nucleation, condensation, and coagulation for the aerosol to reach CCN sizes. In addition, aerosol is diluted while being transported to the cloud. Upon entering the cloud, aerosol particles act as CCN and increase *N*_d_. This leads to a distribution of available condensate to more but smaller droplets and increases their overall surface area and thus cloud albedo. In addition, the microphysical perturbation also affects processes that control the evolution of the macroscopic characteristics of the cloud, in particular precipitation formation, entrainment, local circulations, LWP, cloud fraction, and cloud depth. This discussion can be formalized by the following relationship (Bellouin et al., 2020),

(1) $$\frac{\Delta \alpha }{\Delta \text{ln}N_{\text{a}}}=\frac{\Delta \text{ln}N_{\text{d}}}{\Delta \text{ln}N_{\text{a}}}({\frac{\Delta \alpha }{\Delta \text{ln}N_{\text{d}}}|}_{\text{LWP},C_{\text{F}}}+{\frac{\Delta \alpha }{\Delta \text{ln}\;\text{LWP}}|}_{N_{\text{d},C_{\text{F}}}}\times \frac{\Delta \text{ln}\;\text{LWP}}{\Delta \text{ln}N_{\text{d}}}+{\frac{\Delta \alpha }{\Delta \text{ln}C_{\text{F}}}|}_{N_{\text{d},\text{LWP}}}\frac{\Delta \text{ln}C_{\text{F}}}{\Delta \text{ln}N_{\text{d}}}),$$

where *N*_a_ is the aerosol number concentration, and Δ*α* denotes the change in scene albedo in response to an aerosol perturbation Δ*N*_a_. Here, single-directional difference quotients ((Δ*Y/*Δ*X*)|_*Z*_ ≈ *∂Y/∂X*) are represented as a linear relationship; however, they depend upon meteorological conditions and the background aerosol conditions (Glassmeier et al., 2019). The first term on the right-hand side is the Twomey effect which represents the change in cloud albedo at constant LWP and *C*_F_ while the second and third terms on the right-hand side are LWP and *C*_F_ adjustments. The sign of the LWP change can reverse too, and therefore the joint PDF approach employed by Gryspeerdt et al. (2019a) is a useful methodology for quantifying nonlinear behavior. For warm clouds, the expression simplifies to

(2) $$\frac{\Delta \alpha }{\Delta \text{ln}N_{\text{d}}}=\phi_{\text{atm}}\frac{C_{\text{F}}\alpha_{\text{c}}\left(1-\alpha_{\text{c}}\right)}{3}\times \left(1+\frac{5}{2}\frac{\Delta \text{ln}\;\text{LWP}}{\Delta \text{ln}N_{\text{d}}}+\frac{3\left(\alpha_{\text{c}}-\alpha_{\text{clr}}\right)}{\alpha_{\text{c}}\left(1-\alpha_{\text{c}}\right)}\frac{\Delta \text{ln}C_{\text{F}}}{\Delta \text{ln}N_{\text{d}}}\right),$$

where *ϕ*_atm_ is the transfer function that relates a change in top-of-atmosphere albedo to a change in cloud albedo, which typically takes a value of 0.7 (Diamond et al., 2020); *α*_c_ is the cloudy-sky albedo; and *α*_clr_ is the clear-sky albedo. The complete derivation is described in the Supplement (Sect. S2). Table S1 shows the quantitative values of these cloud properties across diverse laboratories that are used to construct the statistics shown in Figs. 10 and S4 (but using fractional changes instead). *R*_e_, cloud optical thickness, LWP, and *N*_d_ are included where provided in the peer-reviewed publications. The extent to which each of these effects influences the overall cloud albedo is strongly dependent on the specific circumstances: (1) cloud susceptibility, (2) thermodynamic phase, (3) aerosol and precursor emission rate, (4) dilution, (5) methodology and observing system, (6) meteorology, and (7) representativeness. Qualitatively, aerosols increase *N*_d_ and can increase or decrease LWP and *C*_F_. Ultimately cloud and scene-averaged albedo impacts radiation as shown in Fig. 10. Isolated volcanoes and ship tracks exhibit the largest fractional changes in *N*_d_ while the changes in clouds from shipping corridors and global-scale average cloud perturbations exhibit weaker responses by comparison. Differences in the cloud responses are influenced by numerous controlling factors that give rise to the diversity shown in Fig. 10. Each factor is discussed below.

### 4.1 Cloud susceptibility

The background cloud state (namely, *N*_d_) to a large extent determines the specific sensitivities of scene albedo and cloud processes to aerosol perturbations. Twomey (1974) showed that cloud albedo sensitivity to a change in *N*_d_ is largest at low *N*_d_ and cloud albedo of 0.5 (Eq. S4), where the background *N*_d_ and *α* set the strength of the cloud albedo susceptibility as shown by the division by *N*_d_ and confirmed in many field campaigns (Ackerman et al., 2000; Durkee et al., 2000b; Ferek et al., 2000; Lu et al., 2009). While *N*_d_ changes at constant LWP can occur (i.e., the LWP in the polluted clouds is the same as the unpolluted clouds on either side of the track) in ship tracks, it is a relatively rare occurrence (roughly 10 %) in satellite-derived ship track databases (Segrin et al., 2007; Christensen and Stephens, 2012). In a majority of ship tracks, the LWP actually decreases, and in roughly 30 % of tracks the decreases are so large that the cloud albedo becomes dimmer in the polluted clouds (Chen et al., 2012). Similar behavior has been observed in volcano, industry, and fire tracks (Toll et al., 2019). Lower cloud albedo has also been identified in ship tracks from in situ measurements (Chen et al., 2012). This effect is generally attributed to the background meteorology (Sect. 4.6). Nevertheless, cloud susceptibility is a useful construct and could be even more useful with an improved understanding of the relationship between meteorological controlling factors and the terms in Eq. (2), as well as a better understanding of the timescales for LWP adjustments (Glassmeier et al., 2021).

### 4.2 Thermodynamic phase

Decreases in cloud albedo are shown to occur more frequently in polluted clouds when they contain ice particles (Christensen et al., 2014). Cloud albedo in this context averages the cloud albedo retrievals from liquid and ice clouds in satellite imagery over the polluted section of an opportunistic experiment. Higher concentrations of ice in polluted ship track clouds have been identified from several hundred cases using the Cloud-Aerosol Lidar with Orthogonal Polarization (CALIOP). The higher occurrence of ice phase retrievals was hypothesized to be caused by an increase in contact or immersion freezing by the plumes of oceangoing vessels that have higher concentrations of solid species such as calcium, ash oxides of vanadium, nickel, sodium, iron oxides, and other heavy metals (Agrawal et al., 2008) that may serve as effective ice-nucleating particles. The cloud albedo effect is weaker in mixed-phase clouds presumably due to enhanced precipitation occurring by greater amounts of ice particle production causing total water path to decrease via glaciation indirect effects (Lohmann, 2002). The cloud albedo effect may also be weaker because colder and deeper clouds with larger *N*_d_ values are less susceptible than thinner shallower warm boundary layer clouds. As many of the cloud perturbations from volcanic aerosols occur at higher latitudes, careful screening of warm boundary layer clouds must be performed for comparison with other laboratories in warmer regions and deeper investigation into glaciation indirect effects. As the distribution of super-cooled liquid clouds may increase with increasing global mean temperature (Mitchell et al., 1989) and more shipping activity is expected across the Arctic in the future as sea ice extent declines further, the study of glaciation indirect effects will be pivotal for understanding the radiative effects of climate change.

### 4.3 Aerosol emission strength

Cloud perturbations are strongly influenced by the strength of the emissions of gases and particles into the atmosphere, but emission rates are highly variable across laboratories. Passively degassing volcanoes typically emit several orders of magnitude more SO_2_ than an oceangoing vessel. While estimates range from about 5000 td^−1^ at Kīlauea compared to 250 td^−1^ at Mt. Michael in the South Sandwich Islands, both have been shown to produce bright volcano tracks (Gassó, 2008). Like volcanoes, ship emissions also exhibit a wide diversity in emission rates. Measurements from Hobbs et al. (2000) demonstrate that diesel-powered ships burning low-grade marine fuel oil emitted 4–7 times more SO_2_ than gas turbine engines. Brighter, more reflective ship tracks have also been shown to result from ships with higher SO_*x*_ emissions (Gryspeerdt et al., 2019b) or in locations where tracks intersect (Sechrist et al., 2012) but with rapidly diminishing returns related to weaker cloud susceptibility as *N*_d_ increases.

### 4.4 Dilution

Aerosol plumes from ship stacks can overwhelm the ambient CCN by several orders of magnitude at local scales ranging from tens to hundreds of kilometers. Over time the emissions disperse and dilute over broader scales. Due to dilution, the aerosol concentration that reaches the cloud will generally be significantly smaller than at the source. For individual ships the typical area affected is approximately 2500 km^2^ from 250 kg of SO_2_ emissions over a 7 h period (Durkee et al., 2000a). Kabatas et al. (2013) examined dilution in ship tracks as a function of time and found that *R*_e_ increases at a rate of 0.5–1 μm per hour along the polluted portions of ship tracks. This translates to about a 2 μm increase over a distance of 100 km at typical container ship speeds of 24 knots (45 km h^−1^). Durkee et al. (2000a) and Gryspeerdt et al. (2021) found that the change in the width of the ship track over time depends on the background concentration of *N*_d_.

Dilution over larger scales may result in weaker cloud responses. The Holuhraun fissure eruption emitted about 120 kt of SO_2_ per day (at its peak in 2014–2015), an equivalent of 4 times the 28 EU member states emission rates (Gislason et al., 2015; Schmidt et al., 2015). This event led to decreases in *R*_e_ across most of the Norwegian sea (Malavelle et al., 2017). Nevertheless, the volcanic emissions from Holuhruan have a much weaker effect on cloud droplet size and liquid water path (Fig. S4) when compared to isolated volcano track studies (e.g., Gassó, 2008; Toll et al., 2017, 2019). The localized sampling of these highly polluted clouds within volcano or ship tracks and their surrounding cleaner clouds provides a significantly greater contrast in cloud properties compared to studies of aggregated emissions over larger areas (e.g., Holuhruan eruption or shipping corridors in the Peters et al., 2011, and Diamond et al. (2020) analyses). The smaller responses at these larger-scale perturbations shown in Fig. S4 may be the result of dilution. Interestingly, when the fractional *N*_d_ increase (Diamond et al., 2020) is normalized by MERRA-2 sulfate perturbation, the change in cloud droplet size and liquid water path is similar to other studies (McCoy et al., 2018; Gryspeerdt et al., 2019b).

Cloud perturbations near the emission sources are likely caused by primary aerosols (e.g., sulfate aerosols formed inside of the smoke stack or black/organic carbon from combustion). Farther away from emission sources or on a larger scale, secondary aerosols (e.g., sulfate aerosols formed from atmospheric transformation of SO_2_) probably become more important (and contribute towards the “background” aerosol state), while a larger fraction of larger-sized primary aerosols may be lost due to wet/dry deposition. Finally, weaker but more widespread effects due to greater dilution could also lead to greater overall reflection of sunlight since the Twomey effect is sublinear and the polluted clouds may deepen and cover a larger region. Overall, the extent to which the magnitudes of cloud responses across these studies are influenced by dilution and whether the responses can be normalized by some other means for a comparative study remain open research questions.

### 4.5 Methodology and observing system

Methodology and observing systems (in situ, satellite, and modeling) and spatiotemporal scale have been shown to influence ACI metrics (McComiskey and Feingold, 2012). The biases in coarse-scale models are likely related to parameterized physics and unresolved/missing processes (such as entrainment feedbacks). If there is a mismatch in the spatiotemporal scale of the perturbation in relation to the scale of the observing system, then attributing aerosol–cloud interactions in a diluted manner (space or time) is also likely to induce biases (Kabatas et al., 2013; Possner et al., 2016; Gryspeerdt et al., 2021; Glassmeier et al., 2021). The latter may be more of an issue for ship and industrial tracks than larger-scale volcanic eruptions. In the case of volcanoes, biases would be reduced to issues of representativeness of local measurements in an inhomogeneous field rather than scale mismatches (for a more complete discussion of representation error see Schutgens, 2020). Scale mismatches will also need to be considered if the scaling of results from plumes of differing degrees of dilution is to be attempted (see Sect. 4.4 above).

Possner et al. (2015) demonstrated that a regional model running with a 2 km grid spacing was able to capture the structure of an observed ship track. They demonstrated that the ship emissions generated a doubling of the cloud optical thickness, an increase in *N*_d_ by 300 %, and decrease in *R*_e_ by about 40 %. In addition, Possner et al. (2016) studied the dependency of the clouds’ response to ship emissions on the model resolution. They used a regional model at a range of resolutions, ranging from a GCM scale (*D*_*x*_ = 50 km) to the convection-resolving scale (*D*_*x*_ = 1 km), to assess the impact of emission dilution and mixing of aerosols in the atmosphere. They demonstrated that both processes contributed almost equally to the simulated increase in the shortwave cloud radiative effect at coarser (50 km) horizontal resolution. The contrast in the aerosol radiative effect across model resolutions suggests more closure studies are needed to resolve this gap.

Cloud sensitivity to ship emissions on a larger, more climate relevant, scale is estimated using GCMs. For example, Lauer et al. (2007) used a GCM to study the impact of particulate matter from ship emissions on aerosols, clouds, and the radiation budget under different emission inventories. They demonstrated that emissions from ships increased the area mean *N*_d_ of low marine clouds by up to 30 % depending on the geographic region, while the change in liquid water content was small. In addition, the *R*_e_ values were shown to decrease, leading to an increase in cloud optical thickness of up to 5 %–10 %, again, depending on the geographical region. Jin et al. (2018) used a GCM to show that the cloud response to ship emissions depended on the natural dimethyl sulfide (DMS) emissions, which determine the background aerosol concentration. In addition, they estimated the global net cloud radiative effect of ship emissions to be −0.153 Wm^−2^. GCMs were also used to study the effect of the 2014–2015 Holuhraun effusive eruption (referred to as the Nornahraun eruption in the paper, which was the unofficial name at the time) on the climate system by Gettelman et al. (2015). They estimated that emissions from the Holuhraun eruption in Iceland resulted in a regional radiative forcing of −0.21 Wm^−2^, 80 % of which was attributed to ACI. These GCM simulations demonstrated that had this level of emissions occurred in summer rather than in autumn, the radiative forcing would have been much larger (−0.61 Wm^−2^, 94 % of which attributable to ACI). During summer the radiative effects are larger due to a greater solar flux and a higher burden of sulfates from gas-phase oxidation.

Uncertainties that can influence the estimate of satellite-retrieved ERFaci are the humidification of aerosols and enhanced reflectance due to scattering off the edges of clouds typically leading to larger estimates of the Twomey effect and adjustments in *C*_F_ (Gryspeerdt et al., 2016; Christensen et al., 2017). Furthermore, invalid assumptions on adiabaticity for non-plane-parallel clouds where 1D radiative transfer is used on 3D clouds can typically result in uncertainties in retrieved *N*_d_ typically larger than 70 % (Grosvenor et al., 2018). One should keep in mind that satellite studies of LWP adjustment suffer from uncertainties that enter into satellite-retrieved values of $\frac{\text{d}\;\text{ln}\;\text{LWP}}{\text{d}\;\text{ln}\;N_{\text{d}}}$ via the retrieval uncertainties in *N*_d_ (Grosvenor et al., 2018). Further uncertainty then follows by different choices made during the quality checks applied to *N*_d_ retrievals. This is exemplified by inconsistent estimates of $\frac{\text{d}\;\text{ln}\;\text{LWP}}{\text{d}\;\text{ln}\;N_{\text{d}}}$ in the subtropical stratocumulus regions (Michibata and Suzuki, 2020; Gryspeerdt et al., 2019a; Possner et al., 2020). These estimates stem from the same retrievals. Yet, different choices made across the three studies in how to address the uncertainty in *N*_d_ lead to a considerable variability in both magnitude and sign of $\frac{\text{d}\;\text{ln}\;LWP}{\text{d}\;\text{ln}\;N_{\text{d}}}$. Such uncertainties in satellite retrievals and differing methods of filtering clouds are also possible explanations for the observation that the LWP adjustments observed in Diamond et al. (2020) are comparable to those of the ship track work of Gryspeerdt et al. (2019a) and Toll et al. (2019) for similar background values of *R*_e_ and *N*_d_.

Finally, more attention should be paid to potential changes in the width of the droplet size distribution (DSD) (Liu and Daum, 2002), which cloud chamber experiments suggest could be quite important (Chandrakar et al., 2016, 2018). If both the width and center of the droplet size distribution are of first-order importance, it may be more useful to think about primary indirect effects (traditional Twomey effect plus narrowing) and secondary indirect effects (adjustments to the DSD shift) rather than adjustments being due to the Twomey/first indirect effect of a larger number (zeroth moment of the DSD) and smaller effective radius (ratio of third and second moments) alone. Some evidence of a modification of the DSD width may be responsible for creating negative biases in the LWP retrievals within the first 100 km of ship tracks where LWP changes are expected to be zero as there would not have been enough time to modify the clouds (Gryspeerdt et al., 2021). Feingold and Siebert (2009) showed the contrasting role of DSD width. When spectral broadening is associated with increasing *N*_d_ (because of competition for water vapor in the relatively polluted, condensation-dominated regime), albedo susceptibility is diminished, whereas when broadening is associated with a reduction in *N*_d_ (the cleaner, coalescence-dominated regime), susceptibility is enhanced. Polarimeter measurements can provide an estimate of the DSD width (e.g., POLDER, over a limited spatial scale) and would be useful additions (e.g., the upcoming NASA Atmosphere Observing System mission or airborne polarimetry) to the observational toolbox.

### 4.6 Meteorology

**Figure 11.**
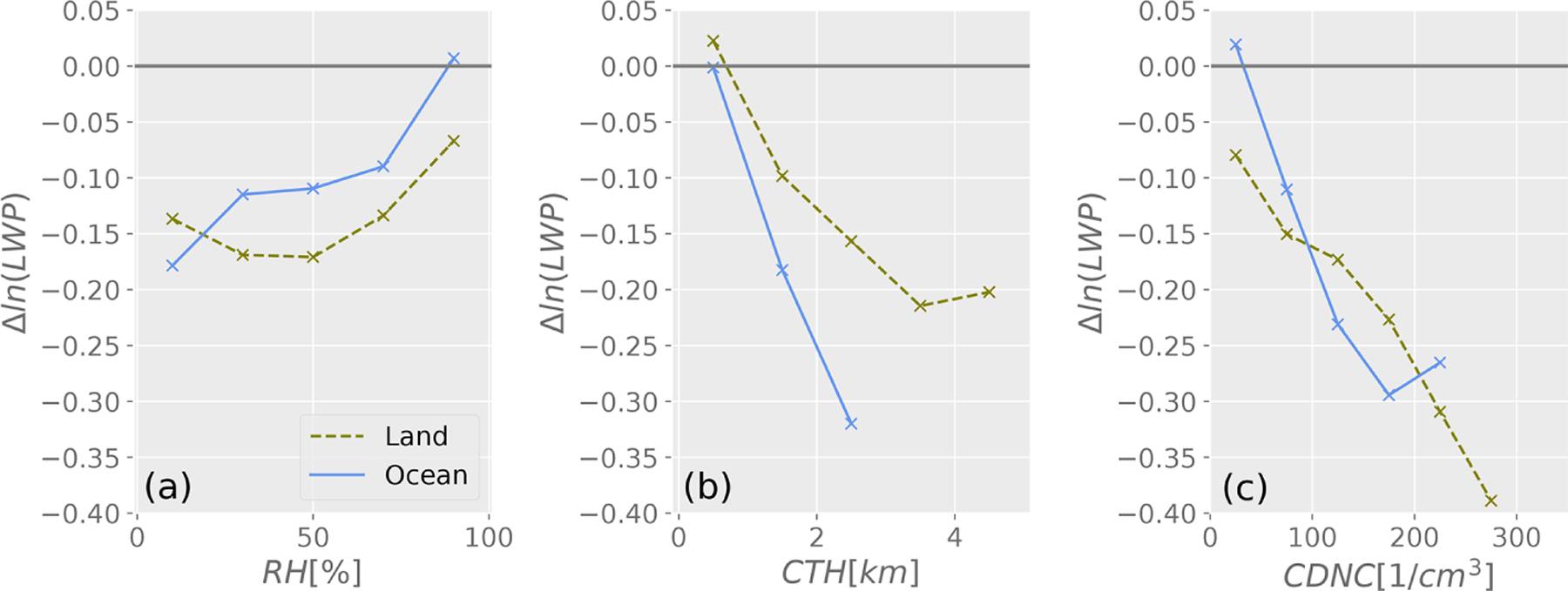
Cloud water response depends on the meteorological conditions. Dependence on above-cloud relative humidity (RH, **a**), cloud top height (CTH, **b**), and background cloud droplet number concentration (*N*_d_, **c**) is shown independently for ocean-based ship and volcano tracks (blue line represents data from Christensen and Stephens, 2012; Toll et al., 2017) and land-based industry and fire tracks (green line represents data from Toll et al., 2019).

The meteorological and aerosol background conditions determine the cloud regime and the processes that dominate cloud evolution. Figure 11 shows the dependence of cloud water response on the environmental conditions for the ocean-based and land-based polluted cloud tracks. The depth of the PBL and free tropospheric humidity have been identified as playing significant roles in the strength of the aerosol–cloud metrics shown in Fig. S4. As the humidity in the free troposphere (above the cloud tops) becomes drier, polluted clouds with smaller droplets evaporate more efficiently (Ackerman et al., 2004; Dagan et al., 2017), causing liquid water paths and cloud albedo to decrease (Coakley and Walsh, 2002; Christensen and Stephens, 2011; Chen et al., 2012; Toll et al., 2019; Gryspeerdt et al., 2021) (Fig. 11). Also, the sign of LWP adjustments $\frac{\text{d}\;\text{ln}\;\text{LWP}}{\text{d}\;\text{ln}\;N}$ is positive when cloud evolution is dominated by precipitation suppression and negative when dominated by evaporation and entrainment. However, precipitation suppression also leads to greater turbulent kinetic energy and more entrainment, and the LWP increase by drizzle suppression (Albrecht, 1989) may only be active when precipitation reaches the surface (Wood, 2007). Figure 11 shows clear cloud water response dependence on baseline *N*_d_: more pristine clouds are more likely to be precipitating, and thus cloud water is more likely to increase.

### 4.7 Representativeness

Four other important challenges for applying lessons learned from natural laboratories and experiments to the study of aerosol–cloud interactions more broadly pertain to representativeness in terms of perturbation concentration, timescale, sampling, and environment.

#### 4.7.1 Perturbation concentration

Concentrated aerosol plumes surrounded by “clean” air behave fundamentally differently than the same amount of aerosol spread out more evenly. Models of isolated ship-track-like plumes show that such concentrated aerosol perturbations can create a secondary circulation transverse to the track. The circulation results in moisture convergence into the track and a positive LWP adjustment and cloud-free downdrafts alongside the track (Wang and Feingold, 2009; Wang et al., 2011). The extent of cloud horizontal clearing along the edges of ship tracks has been shown to buffer the net cloud albedo effect in some ship tracks (Porch et al., 1990). These non-local effects may lead to the overall scene albedo change for an isolated perturbation to differ systematically from what would be obtained by a more uniform increase.

#### 4.7.2 Timescales

More recently, Glassmeier et al. (2021), hereafter G21, have argued that ship track studies underestimate climatological liquid water path decreases from aerosol injections into non-precipitating clouds because evaporation–entrainment adjustments take place on timescales of ~ 20 h. G21 argue that clearly visible ship tracks only persist for ~ 6–7 h and are on average sampled within 3 h of forming and thus do not last long enough to develop substantially negative liquid water adjustments. The results from Diamond et al. (2020) show a more negative liquid water path adjustment which G21 explain as resulting from a longer effective lifetime of ship tracks in the corridor methodology of ≥ 9 h. Thus, the analysis of G21 suggests that short timescale adjustments observed in ship track studies may be unrepresentative of the climatological response to greater aerosol/cloud droplet number. Wood (2007) used mixed layer modeling to show that clouds can thin on short timescales (e.g., when the lifted condensation level rises more quickly than the inversion height) and thicken on longer ones. The adjustment timescale of G21 in fact falls in between these two timescales of an individual stratocumulus cloud system because when quantifying adjustments it compares perturbed and unperturbed systems, each of which have a different equilibration time. The added complexity here is that cloud adjustments seem to vary with time after emissions, and thus near-source impacts are not sufficient for estimating global impacts. The lifetime of industry tracks has not been well quantified. It is unclear whether industry tracks live longer than ship tracks and whether these opportunistic experiments are more representative of the climatological cloud responses (Toll et al., 2019). All of these studies point to the need to account for, and quantify, the timescales of emissions and cloud adjustments for both local and climatically relevant conditions.

#### 4.7.3 Sampling and over-representation

Although Toll et al. (2019) and Trofimov et al. (2020) have made great strides in extending the study of ship-track-like perturbations to deeper continental boundary layers, it remains true that the special cases of shallow well-mixed marine boundary layer with low background aerosol concentrations are over-represented in the natural experiment literature due to the formation of clearly discernible tracks in such environments (Durkee et al., 2000b). However, real but less easily detectable effects may exist in other conditions (Possner et al., 2018), and different integrated aerosol–cloud responses are expected between shallow well-mixed marine boundary layers, deeper decoupled marine boundary layers, and continental boundary layers (Possner et al., 2020). The shipping corridor approach of Diamond et al. (2020) partially addressed this concern by capturing all shipping effects over a defined region from the “top down” rather than building up statistics of clearly detected cases from the “bottom up”. Improved approaches for the detection of pollution tracks via machine learning (Yuan et al., 2019) and trajectory analysis from known point sources (Gryspeerdt et al., 2019a), taken by the ACRUISE project, also provide the opportunity to better sample a more diverse set of regimes via natural experiment methods.

#### 4.7.4 Environmental representativeness

Altogether, the challenges raised above point to the necessity of coupling insights from both modeling and observations even for the seemingly straightforward case of natural experiments like clearly visible ship tracks, in order to extrapolate from the specific situations in which natural experiments can be studied to aerosol–cloud interactions more broadly. The spatial extrapolation of opportunistic experiments requires a good understanding of the dependence of cloud response not only to cloud regime (stratocumulus, shallow cumulus, etc) and dominant microscopic processes (rain- or entrainment-dominated; warm, ice, or mixed phase) but also to external cloud-controlling factors like above-cloud humidity and the typical persistence time of the perturbation. Climate model intercomparisons in specific geographic and meteorological natural experiment settings (Malavelle et al., 2017) could help to overcome the limited representativeness of natural experiments.

## 5 Summary

Experiments of opportunity have been looked upon by some as akin to a “Rosetta Stone” connecting the effects of changing aerosol over the ocean and cloud albedo effects on climate (Porch et al., 1990). It could be argued that ship, volcano, and industrial pollution tracks are the most striking examples of aerosol–cloud interactions in the climate system. A wealth of field campaigns, satellite observations, and modeling studies related to these opportunistic experiments provide incontrovertible evidence that changes in aerosol concentration can lead to significant changes in the microphysics and macrophysics of clouds for the same meteorological conditions. Over the decades, several well-known field campaigns have made a concerted effort to pin down controlling factors that lead to large uncertainty in cloud responses and aerosol indirect radiative forcing as a whole.

Natural laboratories are excellent for process-level understanding of aerosol–cloud interactions. One key result from the Monterey Area Ship Track (MAST) experiment revealed that the cloud condensation nuclei from individual ships are solely responsible for the reflectance perturbations in ship tracks as opposed to the hypotheses involving heat and moisture from the exhaust or sea salt produced in the wake of a ship (Durkee et al., 2000b). While this connection between the aerosol and cloud microphysics is understood, macrophysical responses (such as cloud liquid water path, geometrical thickness, precipitation, and fractional coverage) exhibit more diversity and are poorly understood. Several hypotheses have emerged to explain the bidirectional response in macrophysical responses, and a greater understanding has emerged in recent decades. The dryness of free-tropospheric air can lead to greater evaporation in polluted clouds, thereby decreasing liquid water path (Coakley and Walsh, 2002; Ackerman et al., 2004; Christensen et al., 2009; Chen et al., 2012; Toll et al., 2019; Gryspeerdt et al., 2021). The evolution of the clouds and duration over which they have been influenced by aerosols can affect precipitation, circulation, and liquid water path (Wang and Feingold, 2009; Gryspeerdt et al., 2021).

It remains unclear how representative opportunistic experiments are for understanding of the global response of clouds to anthropogenic aerosols. Typically only shallow clouds are within reach of the emissions from underlying ships or industrial sources, and the albedo cloud susceptibility typically becomes weaker as the PBL deepens (Chen et al., 2012). Furthermore, enhanced lightning in shipping lanes may suggest deep convective clouds are also influenced by shipping aerosol (Thornton et al., 2017). Thus, it is unclear how reliable extrapolations of these opportunistic experiments are to the global scale. The timescale of cloud perturbations is one key aspect of these extrapolations for quantifying global aerosol radiative forcing (Glassmeier et al., 2021). Furthermore, satellite observations typically focus on the “hits” where tracks are observed instead of the “misses” where aerosols may influence clouds but not produce an evident track. The extent to which deeper clouds respond to dilute plumes and radiative forcing remains largely unanswered. It has been estimated that the global coverage of ship tracks is only 0.002 % (Schreier et al., 2007). In order to accurately determine the global ERFaci a new framework may be needed to track individual plumes through to cloud responses when “tracks” are not directly observed by our current and planned observing systems.

This review paper collates the results from experiments of opportunity in over 50 publications. These experiments can provide useful observational constraints on ERF_aci_ through the quantification of key terms represented in Eq. (2). Figures 10 and S4 show good agreement of the increases in cloud droplet number concentration (Fig. 10a) and decreases in cloud droplet effective radius associated with most opportunistic experiments (Fig. 10b). The larger-scale assessments of corridors (satellite) or global shipping (simulations) have smaller drop number perturbations, perhaps indicating dilution effects. There is less agreement on the sign on the LWP response, with uncertainties typically spanning a wide range of negative and positive values (Fig. 10c). Observations of tracks see decreases in LWP, while models tend to show increases, and corridor observations are mixed. This analysis provides a hint that different adjustment processes dominate on different space and timescales. This approach which combines opportunistic experiments may offer a useful framework for future studies, as it is essential to pin down LWP and *C*_F_ adjustments for more accurate estimates of ERFaci. The range of uncertainty in these ACI metrics denotes the important roles of several cloud controlling factors. Two field campaigns, ACTIVATE and ACRUISE, have recently shifted the focus from individual plume-scale cloud interactions to larger regional- and global-scale perturbations to better characterize dilution and nonlinear cloud responses as they relate to emission strength. Furthermore, a better understanding of aerosol’s invigoration of convective and ice clouds alongside the temporal evolution as the clouds evolve and change in accordance with meteorology is essential to understand the albedo responses as they relate to macrophysical cloud property changes. Coordinated model experiments, such as AeroCom have been instrumental in pinpointing deficiencies in atmospheric models and their diversity of simulated effective aerosol radiative forcing (Malavelle et al., 2017).

Finally, opportunistic experiments may assist in understanding large-scale sulfate injection or marine cloud brightening for geoengineering. They might be used to better understand potential geoengineering pathways in similar or analogous environments where the environmental impacts can be quantified (National Academies of Sciences, 2021). Many natural laboratories cause low-cloud perturbations and may well serve as useful analogs for developing climate intervention strategies, so understanding them is critical for future and past aerosol radiative forcing.

## Supplementary Material

SI
