# Supplementary material for: Opportunistic experiments to constrain aerosol effective radiative forcing
Source: Atmos Chem Phys. Author manuscript; Available in PMC 2022 Feb 7. (PMC8819675; doi:10.5194/acp-22-641-2022)
Supplement: SI [file NIHMS1772664-supplement-SI.pdf]

Supplement of Atmos. Chem. Phys., 22, 641–674, 2022
https://doi.org/10.5194/acp-22-641-2022-supplement
© Author(s) 2022. CC BY 4.0 License.

Atmospheric Chemistry and Physics
Open Access

Supplement of

Opportunistic experiments to constrain aerosol effective radiative forcing

Matthew W. Christensen et al.

Correspondence to: Matthew W. Christensen (matt.christensen@pnnl.gov)

The copyright of individual parts of the supplement might differ from the article licence.

1 Contents of this file:

- Text S1 – S2
- Figure S1 – S4
- Tables S1 – S4

2 Text S1

The analysis of CESM2 using the kriging method in Figures 10 and Figure S2 follows Diamond et al. (2020) except in the following ways. The tropical and subtropical domains are defined as spanning (12.5 °W to 2.5 °E, 9.9 to 2.4 °S) and (7.5 °W to 7.5 °E, 18.4 to 10.8 °S), respectively. The center of the shipping affected region is selected by finding the maximum value of sulfate, rather than sulfur dioxide, emissions and then selecting that grid box plus two on either side for each latitude. The covariates for fitting the mean function include lower tropospheric stability (LTS; calculated as the difference in potential temperature between 992 and 773 hPa) but not the "effective" LTS measure taking into account advection. Unlike in Diamond et al. (2020), no transformation is used for the surface sulfate data whereas a logarithmic transformation is used for the cloud droplet number concentration data when fitting the model used for kriging. The analyses shown are for the austral spring (September-October-November) climatology from 2003-2015 in Diamond et al. (2020) and from simulated years 2017-2028 for CESM2.

3 Text S2

The change in scene albedo (α) with cloud droplet concentration (N_d) for warm liquid phase clouds (i.e. Equation 2) can be derived assuming the scene albedo is influenced by the coverage of clouds (f_c) with associated cloud albedo (α_c) and surface albedo (α_{clr}) following

$$\alpha = (1 - f_c)\alpha_{clr}\phi_{atm} + \alpha_c\phi_{atm}f_c \tag{1}$$

where ϕ_{atm} is the transfer function that accounts for the average albedo of the air above the surface and clouds and takes an average value of 0.7 (Diamond et al., 2020), α_c can be estimated using the two-stream delta Eddington approximation assuming the surface albedo beneath the cloud is zero as

$$\alpha_c = \frac{(1-g)\tau_c}{2+(1-g)\tau_c} \tag{2}$$

where g is the asymmetry parameter and takes a value of 0.85 for warm cloud and τ_c is the cloud optical thickness which is approximated using an adiabatic assumption as $\tau_c = \gamma^p L^{\frac{5}{6}} N_d^{\frac{1}{3}}$ where γ^p is a constant value of 1.37e-5 m$^{-0.5}$, L is the liquid water path and N_d is the cloud droplet concentration. Taking the derivative of α with respect to N_d gives

$$\frac{d\alpha}{dN_d} = \phi_{atm}\left(-\alpha_{clr}\frac{\partial f_c}{\partial N_d} + \alpha_c\frac{\partial f_c}{\partial N_d} + f_c\frac{\partial \alpha_c}{\partial N_d}\right) \tag{3}$$

where cloud-free conditions give $\frac{\partial \alpha_{clr}}{\partial N_d} = 0$. The chain rule expansion of $\frac{d\alpha_c}{dN_d} = \frac{\partial \tau_c}{\partial N_d}\frac{\partial \alpha_c}{\partial \tau_c}$ can be solved by the following two derivatives: 1) $\frac{\partial \tau_c}{\partial N_d} = \frac{\tau_c}{3N_d}\left(1+\frac{5}{2}\frac{\partial \ln N_d}{\partial \ln L}\right)$ and 2) $\frac{\partial \alpha_c}{\partial \tau_c} = \frac{\alpha_c(1-\alpha_c)}{\tau_c}$. Combining with equation (3) gives the resulting equation

$$\frac{d\alpha}{dN_d} = \phi_{atm}\frac{f_c\alpha_c(1-\alpha_c)}{3N_d}\left(1+\frac{5}{2}\frac{\partial \ln L}{\partial \ln N_d} + \frac{3(\alpha_c-\alpha_{clr})}{\alpha_c(1-\alpha_c)}\frac{\partial \ln f_c}{\partial \ln N_d}\right) \tag{4}$$

which, after substitution of partial derivatives to finite changes, is equation 2 in the manuscript.

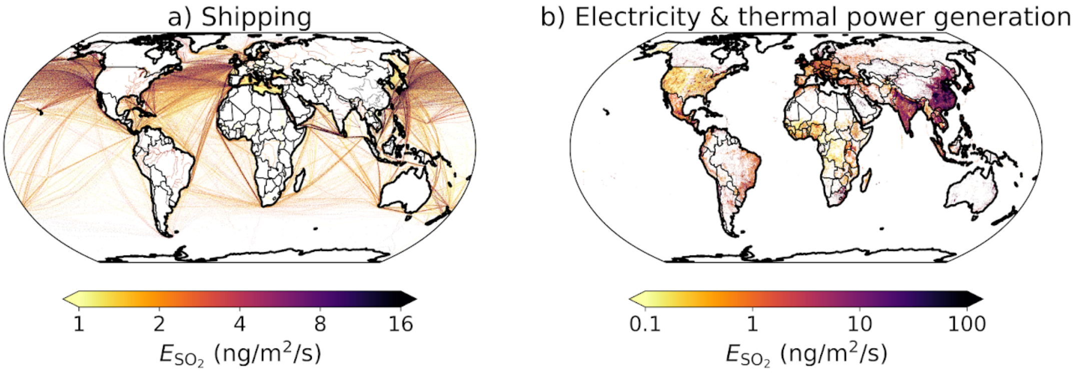

Figure S1. Sulfur dioxide emissions for 2015 from EDGAR for a) shipping and b) the power industry and combustion for manufacturing.

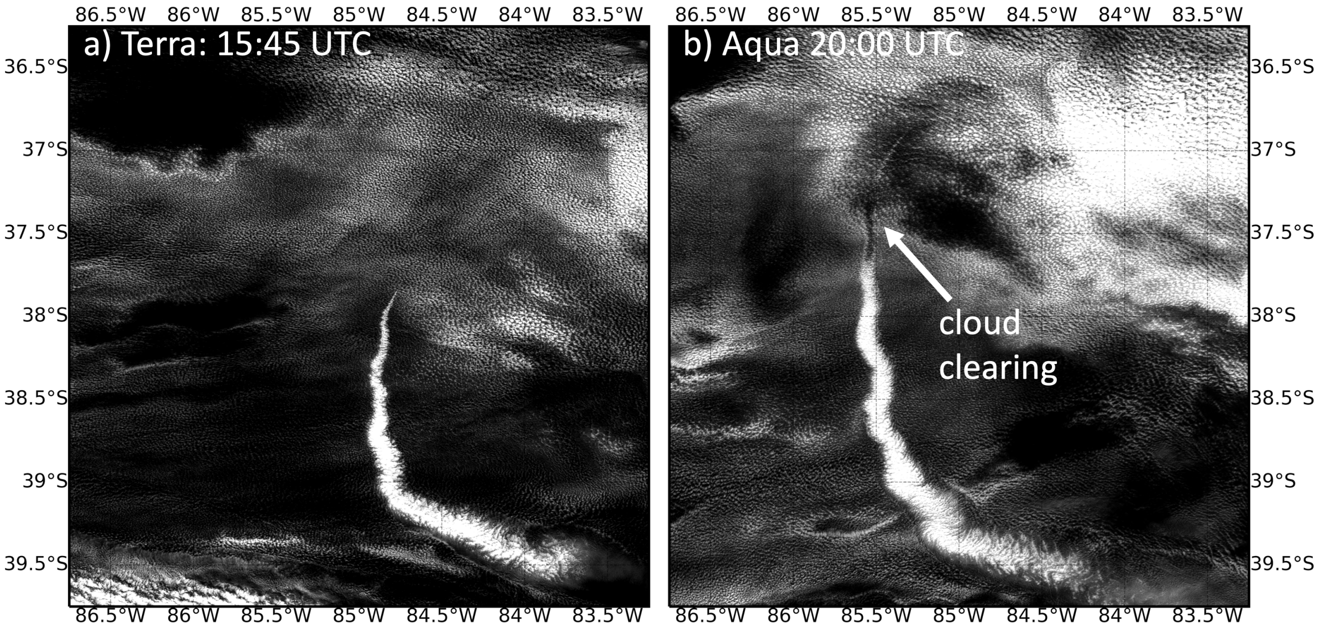

Figure S2. Visible image (0.64-μm reflectance) of ship track captured in MODIS 250 m pixel-scale resolution imagery from the Terra (a) and Aqua (b) satellites on 08/22/06.

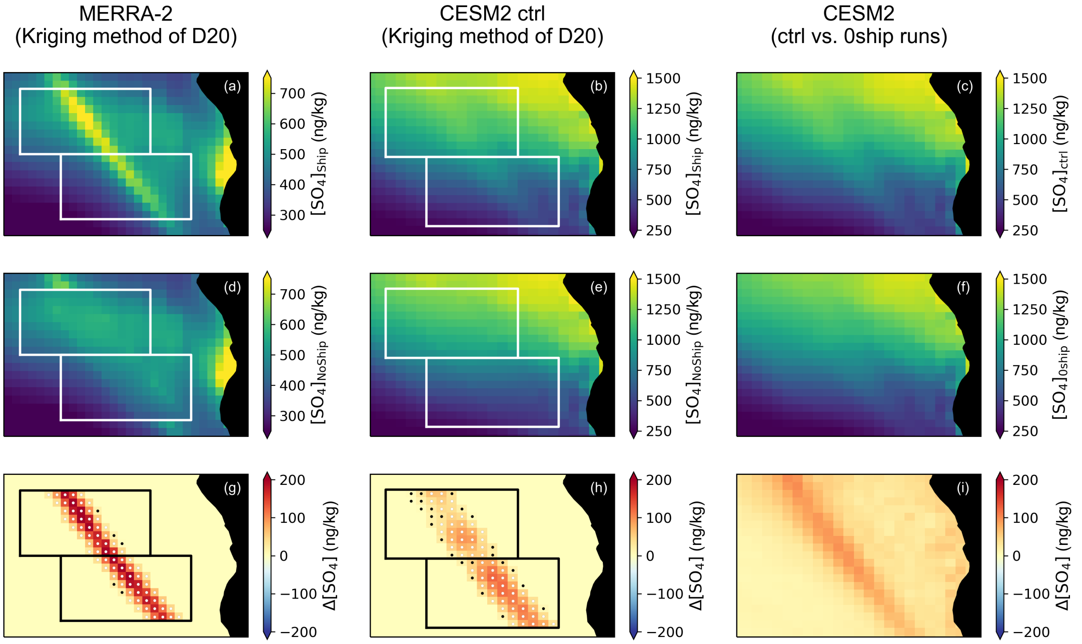

Figure S3. Comparison of Diamond et al. (2020) shipping corridor results for surface sulfate mass concentration with CESM2 output. Factual ("Ship") fields for a) MERRA-2 and b-c) CESM2 control ("ctrl"); counterfactual ("NoShip") fields obtained by kriging for d) MERRA-2 and e) CESM2 ctrl and f) results from CESM2 with zero shipping emissions ("0ship"); and the g-h) factual-counterfactual or i) ctrl-0ship differences. For panels g-h), white dots indicate significance at 95% confidence whereas black dots indicate values that are not statistically significant.

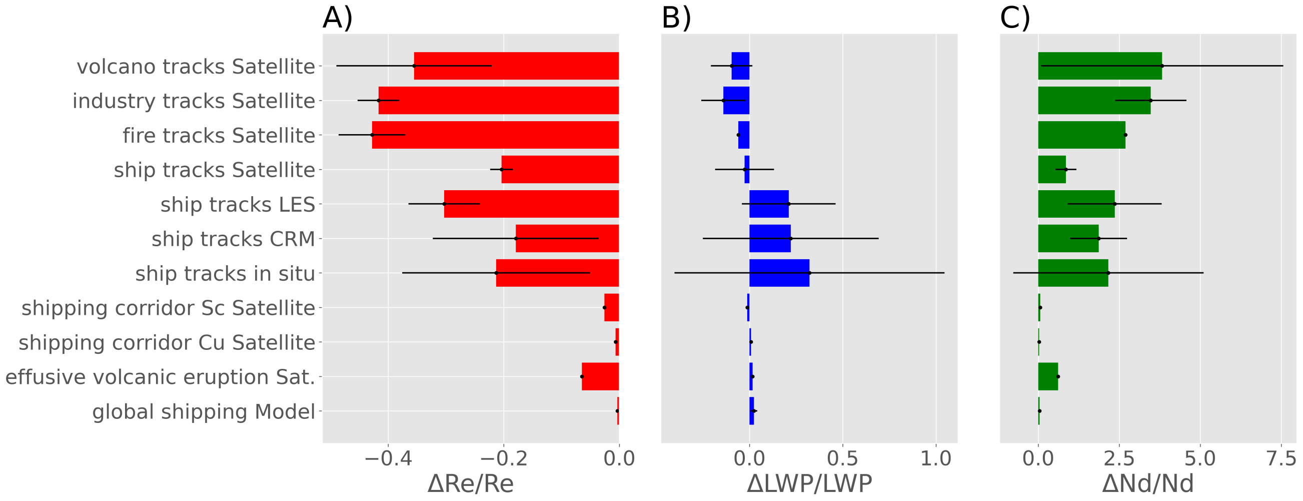

Figure S4. Fractional change in a) effective droplet radius ($\Delta R_e/R_e$), b) liquid water path (ΔLWP/LWP) and c) droplet number concentration ($\Delta N_d/N_d$) averaged over numerous studies involving experiments of opportunity. The number of studies going into each category are as follows: volcano tracks Satellite (6), industry tracks Satellite (9), fire tracks Satellite (1), ship tracks Satellite (9), ship tracks LES (6), ship tracks CRM (4), ship tracks in situ (18), shipping corridor Sc Satellite (2), shipping corridor Cu Satellite (2), effusive volcanic eruption Sat. (1), global shipping Model (3). For a complete listing see Table S1. Error bars represent one standard deviation of reported values for each category representing diversity of the mean amongst studies.

Table S1: List of experiments of opportunity

Author	Laboratory	Data	Regime	Location	ΔNd/Nd	ΔRe/Re	ΔLWP/LWP
Ackerman et al. (2000)	sTracks	in situ	Sc	NEPAC		-0.33	-0.12
Ackerman et al. (2000)	sTracks	in situ	Sc	NEPAC		-0.20	0
Berner et al. (2015)	sTracks	LES	Sc	NEPAC(perp)	1.80	-0.24	0.56
Berner et al. (2015)	sTracks	LES	Sc	NEPAC(base)	3.71	-0.38	0.25
Berner et al. (2015)	sTracks	LES	Sc	NEPAC(iAer)	1.24	-0.25	-0.10
Chen et al. (2012)	sTracks	in situ	closed	NEPAC	0.47	-0.11	-0.33
Chen et al. (2012)	sTracks	in situ	closed	NEPAC	1.12	-0.17	0.62
Chen et al. (2012)	sTracks	in situ	closed	NEPAC	0.26	-0.06	-0.17
Chen et al. (2012)	sTracks	in situ	open	multi	1.79	-0.05	2.82
Christensen and Stephens (2011)	sTracks	Satellite	open	NEPAC	1.50	-0.22	0.39

Continued on next page

Table S1 – *Continued from previous page*

Author	Laboratory	Data	Regime	Location	ΔNd/Nd	ΔRe/Re	ΔLWP/LWP
Christensen and Stephens (2011)	sTracks	Satellite	closed	NEPAC	0.70	-0.17	-0.07
Christensen et al. (2009)	sTracks	Satellite	Sc	NEPAC	0.95	-0.21	-0.08
Christensen et al. (2009)	sTracks	Satellite	Sc	NEPAC	0.86	-0.19	-0.04
Christensen et al. (2014)	sTracks	Satellite	Liq	multi	0.94	-0.20	-0.02
Christensen et al. (2014)	sTracks	Satellite	Mix	multi	0.86	-0.21	-0.16
Coakley and Walsh (2002)	sTracks	Satellite	Sc	NEPAC	1.10	-0.24	-0.18
Diamond et al. (2020)	sCorridor	Satellite	Sc	SEATL(sub)T	0.07	-0.03	-0.006
Diamond et al. (2020)	sCorridor	Satellite	Sc	SEATL(sub)A	0.05	-0.03	-0.02
Diamond et al. (2020)	sCorridor	Satellite	Cu	SEATL(trp)T	0.04	-0.007	0.01
Diamond et al. (2020)	sCorridor	Satellite	Cu	SEATL(trp)A	0.02	-0.006	0.002
Ebmeier et al. (2014)	vTracks	Satellite	Cu	Kilauea	3.16	-0.38	-0.21
Ebmeier et al. (2014)	vTracks	Satellite	Cu	Yasur	1.21	-0.22	0.07
Ebmeier et al. (2014)	vTracks	Satellite	Cu	Piton	0.71	-0.16	0.03
Ferek et al. (1998)	sTracks	in situ	St	NEPAC	1.60	-0.33	-0.36
Ferek et al. (2000)	sTracks	in situ	St	NEPAC	10.2	-0.53	0.41
Ferek et al. (2000)	sTracks	in situ	St	NEPAC	0.47	-0.12	0
Ferek et al. (2000)	sTracks	in situ	St	NEPAC	8.84	-0.50	0.51
Ferek et al. (2000)	sTracks	in situ	St	NEPAC	3.76	-0.37	0.41
Hobbs et al. (2000)	sTracks	in situ	St	NEPAC	1.88	-0.45	-0.08
Hobbs et al. (2000)	sTracks	in situ	St	NEPAC	0.08	-0.05	
Lauer et al. (2007)	globe ship	Model	Liq	global	0.04	-0.006	0.05
Lu et al. (2009)	sTracks	in situ	closed	multi	0.68	-0.11	0.29
Malavelle et al. (2017)	vEruption	Satellite	Liq	Holuhraun	0.61	-0.07	0.02
Noone et al. (2000)	sTracks	in situ	St	NEPAC	0.08	-0.03	-0.09
Noone et al. (2000)	sTracks	in situ	St	NEPAC	0.02	-0.006	-0.06
Peters et al. (2013)	globe ship	Model	Liq	global	0.03	-1.00e-04	0.006
Peters et al. (2013)	globe ship	Model	Liq	global	0.05	-0.004	0.02
Possner et al. (2015)	sTracks	CRM	St	Europe	3.00	-0.40	1.00e+00
Possner et al. (2017)	sTracks	LES	St	Arctic	2.12	-0.35	0.08
Possner et al. (2018)	sTracks	CRM	St	SEPAC(ctrl)	0.56	0	0.17

Continued on next page

Table S1 – *Continued from previous page*

Author	Laboratory	Data	Regime	Location	ΔNd/Nd	ΔRe/Re	ΔLWP/LWP
Possner et al. (2018)	sTracks	CRM	St	SEPAC(detr)	2.12	-0.17	-0.05
Possner et al. (2018)	sTracks	CRM	St	SEPAC(wall)	1.77	-0.14	-0.23
Radke et al. (1989)	sTracks	in situ	St	NEPAC	1.67	-0.19	1.00e+00
Radke et al. (1989)	sTracks	in situ	St	NEPAC	1.75	-0.23	0.61
Rosenfeld (1999)	fTracks	Satellite	Cu	Indonesia		-0.49	
Segrin et al. (2007)	sTracks	Satellite	St	NEPAC	0.35	-0.19	-0.06
Segrin et al. (2007)	sTracks	Satellite	St	NEPAC	0.44	-0.19	-0.03
Toll et al. (2019)	fTracks	Satellite	St	Russia	2.69	-0.37	-0.06
Toll et al. (2019)	iTracks	Satellite	St	Kazakhstan	2.42	-0.41	-0.41
Toll et al. (2019)	iTracks	Satellite	St	Moscow	2.70	-0.38	-0.22
Toll et al. (2019)	iTracks	Satellite	St	Kalgoorlie	3.03	-0.39	-0.08
Toll et al. (2019)	iTracks	Satellite	St	Labrador	4.00	-0.45	-0.21
Toll et al. (2019)	iTracks	Satellite	St	Newfoundland	3.94	-0.43	0.01
Toll et al. (2019)	iTracks	Satellite	St	Nenets	2.81	-0.39	-0.12
Toll et al. (2019)	iTracks	Satellite	St	Manitoba	2.90	-0.41	-0.10
Toll et al. (2019)	iTracks	Satellite	Liq	Traralgon	3.22	-0.39	-0.02
Toll et al. (2019)	vTracks	Satellite	Cu	Ambrym	11.9	-0.57	-0.19
Toll et al. (2019)	vTracks	Satellite	St	Kuril	2.61	-0.38	-0.17
Toll et al. (2019)	vTracks	Satellite	St	Sandwich	3.34	-0.42	-0.11
Trofimov et al. (2020)	iTracks	Satellite	St	Norilsk	6.23	-0.50	-0.11
Wang et al. (2011)	sTracks	LES	rain	NEPAC	4.76		0.51
Wang et al. (2011)	sTracks	LES	norain	NEPAC	0.52		-0.03

Table S1: List of experiments of opportunity from an expert solicitation of peer-reviewed articles used in Fig. 10. Ship tracks (sTracks), Industry tracks (iTracks), Fire tracks (fTracks), Volcano tracks (vTracks), vEruption (effusive volcanic eruption) are renamed for brevity. Liquid cloud types, Stratus (St), Stratocumulus (Sc), Cumulus (Cu), have top heights greater than 500 hPa (Liq) and mixed (MIX) phase cloud. Northeast Pacific (NEPAC: $\sim 130° - 120°$ W; $20° - 30°$ N), South East Atlantic (SEATL: $\sim 0° - 10°$ E; $20° - 10°$ S), South East Pacific (SEPAC: $\sim 80° - 90°$ W; $20° - 10°$ S), and multiple basins (multi) have shorter names. Diamond et al. (2020) separate subtropical (sub) from tropical (trp) for retrievals from Terra (T), Aqua (A). Multiple LES simulations were performed by Berner et al. (2015) and Possner et al. (2018). The full list can be found on google docs https://docs.google.com/spreadsheets/d/1_xxezi1dMq3-We3zcb1-C6PSMCYmXgm21Ro8niLsErA/edit#gid=980524081.

Database Developer	Type	Region	Period	Key Result	Status
Satellite Observations					
Coakley	Ship tracks (+4,000 hand-logged Terra and Aqua MODIS)	NW Pacific	2002–2004 (summer)	Ship tracks found to have less LWP in MODIS overcast cloud retrievals.	Private – extant (Segrin et al., 2007)
Christensen	Ship tracks (1,600 hand-logged Aqua MODIS collocated to CloudSat)	N Pacific, SE Pacific, SE Atlantic	2006 –2010	Ship tracks in open cells have higher cloud tops retrieved using CALIPSO.	Private – extant (Christensen and Stephens, 2012)
Toll	Industry, volcano and ship tracks (5,629 Hand-logged MODIS)	Russia, Kazakhstan, Australia, Canada, Vanuatu, Kuril Islands, South Sandwich Islands	2002 – 2017	Relatively weak decrease in LWP compared to R_e decrease.	https://doi.org/10.17864/1947.208. (Toll et al., 2019)
Trofimov; Toll	Industry tracks (331 MODIS)	Norilsk, Russia and limited number from other regions	2000 – 2017	Large-scale industrial cloud perturbations confirm bidirectional LWP response.	http://dx.doi.org/10.15155/re-140 (Trofimov et al., 2020)
Gryspeerdt	Ship tracks (+17,000 Hand-logged MODIS)	NW Pacific, NW Atlantic, European ECA region	2003, 2014, 2015, 2016	SECA policy decreases ship track occurrence and brightness.	Private – extant (Gryspeerdt et al., 2019)
Yuan	Ship tracks	Global	2002 – 2020	Machine learning successfully detects ship tracks.	Private – extant (Yuan et al., 2019)
Carn	Network of passive degassing volcano estimates of SO_2 fluxes	Worldwide	2005 – 2020	OMI retrievals to reliably estimate emissions in remote locations.	https://www.nature.com/articles/srep44095 (Carn et al., 2017)
General Circulation Models					
AeroCOM - VolcACI	Holuhruan Fissure eruptions MODIS	N. Atlantic	2014 – 2015	Multimodel simulation of strong Nd response, no LWP response in MODIS.	https://wiki.met.no/aerocom/phase3-experiments (Malavelle et al., 2017)
In Situ					
MAST	Numerous ship tracks sampled from in situ measurements	NW Pacific	1996	Extensive field campaign. Ship tracks are the direct manifestation of aerosols not from heat or wakes.	Private – unknown (Durkee et al., 2000)
MASE	Numerous ship tracks sampled from in situ measurements	NW Pacific	2005	Investigation of microphysics and macrophysics as they relate to the Twomey effect.	Private – unknown (Lu et al., 2009)
E-PEACE	Numerous ship tracks sampled from in situ measurements	NW Pacific	2010	Smoke generators produce ship tracks and cause cooling.	https://www.nature.com/articles/sdata201826
ACRUISE	Shipping corridors and some isolated ship tracks	English Channel & off the Porteguese Coast	2019	Quantify impact of policy change on aerosol composition and cloud properties.	https://www.faam.ac.uk/data-centre/

Table S2. List of observational databases for experiments of opportunity. Note, private - extant datasets mean that they can generally be obtained by request as their known source is identifiable.

Laboratory	ΔRe/Re	ΔLWP/LWP	ΔNd/Nd
volcano tracks Satellite (6)	-0.36 (0.13)	-0.10 (0.11)	3.82 (3.74)
industry tracks Satellite (9)	-0.42 (0.04)	-0.14 (0.12)	3.47 (1.10)
fire tracks Satellite (1)	-0.43 (0.06)	-0.06 (0)	2.69 (0)
ship tracks Satellite (9)	-0.20 (0.02)	-0.03 (0.16)	0.85 (0.32)
ship tracks LES (6)	-0.30 (0.06)	0.21 (0.25)	2.36 (1.45)
ship tracks CRM (4)	-0.18 (0.14)	0.22 (0.47)	1.86 (0.88)
ship tracks in situ (18)	-0.21 (0.16)	0.32 (0.72)	2.16 (2.94)
shipping corridor Sc Satellite (2)	-0.03 (4.50e-04)	-0.01 (0.007)	0.06 (0.01)
shipping corridor Cu Satellite (2)	-0.007 (8.00e-04)	0.009 (0.006)	0.03 (0.008)
effusive volcanic eruption Sat. (1)	-0.07 (0)	0.02 (0)	0.61 (0)
global shipping Model (3)	-0.004 (0.003)	0.02 (0.02)	0.04 (0.009)

Table S3. List of mean fractional changes in cloud properties for combined opportunistic experiments listed in Table S1 (numbers in parenthesis next to each experiment denote the number of peer-reviewed articles which make up the mean and standard deviation). Values in parenthesis denote standard deviations.

Laboratory	ΔlnNd	ΔlnRe/ΔlnNd	ΔlnLWP/ΔlnNd
volcano tracks Satellite (6)	1.37 (0.63)	-0.33 (0.01)	-0.04 (0.09)
industry tracks Satellite (9)	1.54 (0.21)	-0.35 (0.02)	-0.10 (0.10)
fire tracks Satellite (1)	1.36 (0)	-0.34 (0)	-0.07 (0)
ship tracks Satellite (9)	0.66 (0.10)	-0.35 (0.03)	-0.09 (0.18)
ship tracks LES (6)	1.12 (0.44)	-0.32 (0.04)	0.11 (0.19)
ship tracks CRM (4)	1.00 (0.35)	-0.17 (0.13)	0.14 (0.31)
ship tracks in situ (18)	0.83 (0.67)	-0.33 (0.14)	-0.19 (1.04)
shipping corridor Sc Satellite (2)	0.06 (0.010)	-0.42 (0.06)	-0.23 (0.15)
shipping corridor Cu Satellite (2)	0.02 (0.003)	-0.27 (0.004)	0.33 (0.22)
effusive volcanic eruption Sat. (1)	0.21 (0)	-0.32 (0)	0.08 (0)
global shipping Model (3)	0.04 (0.008)	-0.08 (0.06)	0.56 (0.43)

Table S4. List of mean changes in cloud properties (scaled by $\Delta \ln N_d$) for combined opportunistic experiments listed in Table S1 (numbers in parenthesis next to each experiment denote the number of peer-reviewed articles which make up the mean and standard deviation). Values in parenthesis denote standard deviations.

References

Ackerman, A. S., Toon, O. B., Taylor, J. P., Johnson, D. W., Hobbs, P. V., and Ferek, R. J.: Effects of Aerosols on Cloud Albedo: Evaluation of Twomey's Parameterization of Cloud Susceptibility Using Measurements of Ship Tracks, Journal of the Atmospheric Sciences, 57, 2684–2695, https://doi.org/10.1175/1520-0469(2000)057<2684:EOAOCA>2.0.CO;2, 2000.

Berner, A. H., Bretherton, C. S., and Wood, R.: Large Eddy Simulation of Ship Tracks in the Collapsed Marine Boundary Layer: A Case Study from the Monterey Area Ship Track Experiment, Atmospheric Chemistry and Physics, 15, 5851–5871, https://doi.org/10.5194/acp-15-5851-2015, 2015.

Carn, S. A., Fioletov, V. E., McLinden, C. A., Li, C., and Krotkov, N. A.: A Decade of Global Volcanic SO_2 Emissions Measured from Space, Scientific Reports, 7, 44 095, https://doi.org/10.1038/srep44095, 2017.

Chen, Y. C., Christensen, M. W., Xue, L., Sorooshian, A., Stephens, G. L., Rasmussen, R. M., and Seinfeld, J. H.: Occurrence of Lower Cloud Albedo in Ship Tracks, Atmospheric Chemistry and Physics, 12, 8223–8235, https://doi.org/10.5194/acp-12-8223-2012, 2012.

Christensen, M. W. and Stephens, G. L.: Microphysical and Macrophysical Responses of Marine Stratocumulus Polluted by Underlying Ships: Evidence of Cloud Deepening, Journal of Geophysical Research: Atmospheres, 116, https://doi.org/10.1029/2010JD014638, 2011.

Christensen, M. W. and Stephens, G. L.: Microphysical and Macrophysical Responses of Marine Stratocumulus Polluted by Underlying Ships: 2. Impacts of Haze on Precipitating Clouds, Journal of Geophysical Research: Atmospheres, 117, https://doi.org/10.1029/2011JD017125, 2012.

Christensen, M. W., Coakley, J. A., and Tahnk, W. R.: Morning-to-Afternoon Evolution of Marine Stratus Polluted by Underlying Ships: Implications for the Relative Lifetimes of Polluted and Unpolluted Clouds, Journal of the Atmospheric Sciences, 66, 2097–2106, https://doi.org/10.1175/2009JAS2951.1, 2009.

Christensen, M. W., Suzuki, K., Zambri, B., and Stephens, G. L.: Ship Track Observations of a Reduced Shortwave Aerosol Indirect Effect in Mixed-Phase Clouds, Geophysical Research Letters, 41, 6970–6977, https://doi.org/10.1002/2014GL061320, 2014.

Coakley, J. A. and Walsh, C. D.: Limits to the Aerosol Indirect Radiative Effect Derived from Observations of Ship Tracks, Journal of the Atmospheric Sciences, 59, 668–680, https://doi.org/10.1175/1520-0469(2002)059<0668:LTTAIR>2.0.CO;2, 2002.

Diamond, M. S., Director, H. M., Eastman, R., Possner, A., and Wood, R.: Substantial Cloud Brightening From Shipping in Subtropical Low Clouds, AGU Advances, 1, e2019AV000 111, https://doi.org/10.1029/2019AV000111, 2020.

Durkee, P. A., Noone, K. J., and Bluth, R. T.: The Monterey Area Ship Track Experiment, Journal of the Atmospheric Sciences, 57, 2523–2541, https://doi.org/10.1175/1520-0469(2000)057<2523:TMASTE>2.0.CO;2, 2000.

Ebmeier, S. K., Sayer, A. M., Grainger, R. G., Mather, T. A., and Carboni, E.: Systematic Satellite Observations of the Impact of Aerosols from Passive Volcanic Degassing on Local Cloud Properties, Atmos. Chem. Phys., 14, 10 601–10 618, https://doi.org/10.5194/acp-14-10601-2014, 2014.

Ferek, R. J., Hegg, D. A., Hobbs, P. V., Durkee, P., and Nielsen, K.: Measurements of Ship-Induced Tracks in Clouds off the Washington Coast, Journal of Geophysical Research: Atmospheres, 103, 23 199–23 206, https://doi.org/10.1029/98JD02121, 1998.

Ferek, R. J., Garrett, T., Hobbs, P. V., Strader, S., Johnson, D., Taylor, J. P., Nielsen, K., Ackerman, A. S., Kogan, Y., Liu, Q., Albrecht, B. A., and Babb, D.: Drizzle Suppression in Ship Tracks, Journal of the Atmospheric Sciences, 57, 2707–2728, https://doi.org/10.1175/1520-0469(2000)057<2707:DSIST>2.0.CO;2, 2000.

Gryspeerdt, E., Smith, T. W. P., O'Keeffe, E., Christensen, M. W., and Goldsworth, F. W.: The Impact of Ship Emission Controls Recorded by Cloud Properties, Geophysical Research Letters, https://doi.org/10.1029/2019GL084700, 2019.

Hobbs, P. V., Garrett, T. J., Ferek, R. J., Strader, S. R., Hegg, D. A., Frick, G. M., Hoppel, W. A., Gasparovic, R. F., Russell, L. M., Johnson, D. W., O'Dowd, C., Durkee, P. A., Nielsen, K. E., and Innis, G.: Emissions from Ships with Respect to Their Effects on Clouds, Journal of the Atmospheric Sciences, 57, 2570–2590, https://doi.org/10.1175/1520-0469(2000)057<2570:EFSWRT>2.0.CO;2, 2000.

Lauer, A., Eyring, V., Hendricks, J., Jöckel, P., and Lohmann, U.: Global Model Simulations of the Impact of Ocean-Going Ships on Aerosols, Clouds, and the Radiation Budget, Atmospheric Chemistry and Physics, 7, 5061–5079, https://doi.org/10.5194/acp-7-5061-2007, 2007.

Lu, M.-L., Sorooshian, A., Jonsson, H. H., Feingold, G., Flagan, R. C., and Seinfeld, J. H.: Marine Stratocumulus Aerosol-Cloud Relationships in the MASE-II Experiment: Precipitation Susceptibility in Eastern Pacific Marine Stratocumulus, Journal of Geophysical Research: Atmospheres, 114, https://doi.org/10.1029/2009JD012774, 2009.

Malavelle, F. F., Haywood, J. M., Jones, A., Gettelman, A., Clarisse, L., Bauduin, S., Allan, R. P., Karset, I. H. H., Kristjánsson, J. E., Oreopoulos, L., Cho, N., Lee, D., Bellouin, N., Boucher, O., Grosvenor, D. P., Carslaw, K. S., Dhomse, S., Mann, G. W., Schmidt, A., Coe, H., Hartley, M. E., Dalvi, M., Hill, A. A., Johnson, B. T., Johnson, C. E., Knight, J. R., O'Connor, F. M., Partridge, D. G., Stier, P., Myhre, G., Platnick, S., Stephens, G. L., Takahashi, H., and Thordarson, T.: Strong Constraints on Aerosol–Cloud Interactions from Volcanic Eruptions, Nature, 546, 485–491, https://doi.org/10.1038/nature22974, 2017.

Noone, K. J., Johnson, D. W., Taylor, J. P., Ferek, R. J., Garrett, T., Hobbs, P. V., Durkee, P. A., Nielsen, K., Öström, E., O'Dowd, C., Smith, M. H., Russell, L. M., Flagan, R. C., Seinfeld, J. H., De Bock, L., Van Grieken, R. E., Hudson, J. G., Brooks, I., Gasparovic, R. F., and Pockalny, R. A.: A Case Study of Ship Track Formation in a Polluted Marine Boundary Layer, Journal of the Atmospheric Sciences, 57, 2748–2764, https://doi.org/10.1175/1520-0469(2000)057<2748:ACSOST>2.0.CO;2, 2000.

Peters, K., Stier, P., Quaas, J., and Graßl, H.: Corrigendum to "Aerosol indirect effects from shipping emissions: sensitivity studies with the global aerosol-climate model ECHAM-HAM" published in Atmos. Chem. Phys., 12, 5985–6007, 2012, Atmospheric Chemistry and Physics, 13, 6429–6430, https://doi.org/10.5194/acp-13-6429-2013, 2013.

Possner, A., Zubler, E., Lohmann, U., and Schär, C.: Real-Case Simulations of Aerosol–Cloud Interactions in Ship Tracks over the Bay of Biscay, Atmospheric Chemistry and Physics, 15, 2185–2201, https://doi.org/10.5194/acp-15-2185-2015, 2015.

Possner, A., Ekman, A. M. L., and Lohmann, U.: Cloud Response and Feedback Processes in Stratiform Mixed-Phase Clouds Perturbed by Ship Exhaust, Geophysical Research Letters, 44, 1964–1972, https://doi.org/10.1002/2016GL071358, 2017.

Possner, A., Wang, H., Wood, R., Caldeira, K., and Ackerman, T.: The Efficacy of Aerosol-Cloud-Radiative Perturbations from near-Surface Emissions in Deep Open-Cell Stratocumuli, Atmospheric Chemistry and Physics, 18, 17 475–17 488, https://doi.org/10.5194/acp-18-17475-2018, 2018.

Radke, L. F., Coakley, J. A., and King, M. D.: Direct and Remote Sensing Observations of the Effects of Ships on Clouds, Science, 246, 1146–1149, 1989.

Rosenfeld, D.: TRMM Observed First Direct Evidence of Smoke from Forest Fires Inhibiting Rainfall, Geophysical Research Letters, 26, 3105–3108, https://doi.org/10.1029/1999gl006066, 1999.

Segrin, M. S., Coakley, J. A., and Tahnk, W. R.: MODIS Observations of Ship Tracks in Summertime Stratus off the West Coast of the United States, Journal of the Atmospheric Sciences, 64, 4330–4345, https://doi.org/10.1175/2007JAS2308.1, 2007.

Toll, V., Christensen, M., Quaas, J., and Bellouin, N.: Weak Average Liquid-Cloud-Water Response to Anthropogenic Aerosols, Nature, 572, 51–55, https://doi.org/10.1038/s41586-019-1423-9, 2019.

Trofimov, H., Bellouin, N., and Toll, V.: Large-Scale Industrial Cloud Perturbations Confirm Bidirectional Cloud Water Responses to Anthropogenic Aerosols, Journal of Geophysical Research: Atmospheres, 125, e2020JD032 575, https://doi.org/10.1029/2020JD032575, 2020.

Wang, H., Rasch, P. J., and Feingold, G.: Manipulating Marine Stratocumulus Cloud Amount and Albedo: A Process-Modelling Study of Aerosol-Cloud-Precipitation Interactions in Response to Injection of Cloud Condensation Nuclei, Atmospheric Chemistry and Physics, 11, 4237–4249, https://doi.org/10.5194/acp-11-4237-2011, 2011.

Yuan, T., Wang, C., Song, H., Platnick, S., Meyer, K., and Oreopoulos, L.: Automatically Finding Ship Tracks to Enable Large-Scale Analysis of Aerosol-Cloud Interactions, Geophysical Research Letters, 46, 7726–7733, https://doi.org/10.1029/2019gl083441, 2019.
